# Mechano-biochemical marine stimulation of inversion, gastrulation, and endomesoderm specification in multicellular Eukaryota

**DOI:** 10.3389/fcell.2022.992371

**Published:** 2022-12-01

**Authors:** Ngoc Minh Nguyen, Tatiana Merle, Florence Broders-Bondon, Anne-Christine Brunet, Aude Battistella, Emelie Britt Linnea Land, Florian Sarron, Aditya Jha, Jean-Luc Gennisson, Eric Röttinger, María Elena Fernández-Sánchez, Emmanuel Farge

**Affiliations:** ^1^ Mechanics and Genetics of Embryonic Development Group, Institut Curie, Centre OCAV PSL Research University, CNRS, UMR168, Inserm, Sorbonne University, Paris, France; ^2^ Biochemistry, Molecular Biology, and Cells Platform, Institut Curie, CNRS, UMR 168, Inserm, Sorbonne University, Paris, France; ^3^ Sorbonne Université, CNRS, UMR 7095, Institut d'Astrophysique de Paris, Paris, France; ^4^ Laboratoire Physique et Mécanique des Milieux Hétérogènes (PMMH), CNRS, ESPCI ParisTech, Université Pierre et Marie Curie, Université Paris Diderot, Paris, France; ^5^ Université Paris-Saclay, CEA, CNRS, Inserm, BioMaps, Service Hospitalier Frédéric Joliot, Orsay, France; ^6^ Université Côte d’Azur, CNRS, INSERM, Institute for Research on Cancer and Aging (IRCAN), Nice, France; ^7^ Université Côte d’Azur, Institut Fédératif de Recherche Ressources Marines (IFR MARRES), Nice, France

**Keywords:** hydrodynamic mechanical strains, cnidaria *Nematostella vectensis*, choanoflagellate *Choanoeca flexa*, mechanotransduction, evolutionary emergence of first Metazoa organisms, myosin-dependent mechanosensitivity, beta-catenin-dependent mechanosensitivity, primitive motor-sensorial behavioral mechanosensing

## Abstract

The evolutionary emergence of the primitive gut in Metazoa is one of the decisive events that conditioned the major evolutionary transition, leading to the origin of animal development. It is thought to have been induced by the specification of the endomesoderm (EM) into the multicellular tissue and its invagination (i.e., gastrulation). However, the biochemical signals underlying the evolutionary emergence of EM specification and gastrulation remain unknown. Herein, we find that hydrodynamic mechanical strains, reminiscent of soft marine flow, trigger active tissue invagination/gastrulation or curvature reversal *via* a Myo-II-dependent mechanotransductive process in both the metazoan *Nematostella vectensis* (*cnidaria*) and the multicellular choanoflagellate *Choanoeca flexa.* In the latter, our data suggest that the curvature reversal is associated with a sensory-behavioral feeding response. Additionally, like in bilaterian animals, gastrulation in the cnidarian *Nematostella vectensis* is shown to participate in the biochemical specification of the EM through mechanical activation of the β-catenin pathway *via* the phosphorylation of Y654-βcatenin. Choanoflagellates are considered the closest living relative to metazoans, and the common ancestor of choanoflagellates and metazoans dates back at least 700 million years. Therefore, the present findings using these evolutionarily distant species suggest that the primitive emergence of the gut in Metazoa may have been initiated in response to marine mechanical stress already in multicellular pre-Metazoa. Then, the evolutionary transition may have been achieved by specifying the EM *via* a mechanosensitive Y654-βcatenin dependent mechanism, which appeared during early Metazoa evolution and is specifically conserved in all animals.

## Introduction

Metazoa are composed of complex body plans, each defining its distinct functional organs (De Robertis, 2008). The emergence of the first Metazoa organism probably resulted from the evolutionary development of multicellularity, reproduction by egg–sperm fusion, and the formation of the first organ: the primitive gut (Haeckel, 1874; Brunet and King, 2017). The primitive gut was probably formed from primitive multicellular blastulae hollow spheres *via* gastrulation. In today’s early embryos, this process consists of an active morphogenetic movement initiated by the inversion of the geometric curvature of the two-dimensional multicellular leaflet-like tissue (invagination) pre-specified as the endomesoderm (EM) (Arendt, 2004). It remains unclear whether the biochemical signals involved in EM specification and gastrulation are conserved in current early Metazoa embryos. However, the reversal of the curvature in these multicellular leaflets (responsible for the initiation of gastrulation) is regulated by several distinct signaling pathways (EphA4, Fog, and Stb) that depend on the species involved (Sweeton et al., 1991; Kumburegama et al., 2011; Evren et al., 2014). Similarly, the biochemical specification of the EM is regulated by multiple diverse signaling pathways, including Nodal, Dorsal, or Wnts, and is also species-dependent (Roth, 2004; Kusserow et al., 2005; Hagos and Dougan, 2007). Thus, the existence of a common evolutionary origin in the emergence of the primitive digestive organ in Metazoa remains uncertain.

Molecular activation of the Myo-II motor by internal mechanical strains has been shown to trigger invaginations of the mesoderm and endoderm during gastrulation in bilaterian *Drosophila* embryos (Mitrossilis et al., 2017; Bailles et al., 2019)*.* Additionally, mechanical strains were involved in the expression of endodermal and mesodermal genes in early *Drosophila* and zebrafish bilaterian embryos (Desprat et al., 2008; Brunet et al., 2013). Specifically, the mechanical stimulation of Twist and *brachyury* endomesodermal determinants expression was β-catenin (*β*-cat) dependent and stimulated and induced as a result of the mechanical induction of Y654-βcat phosphorylation in response to the early internal morphogenetic movements of embryogenesis, gastrulation, and epiboly, respectively (Desprat et al., 2008; Brunet et al., 2013). Furthermore, the expression of *brachyury* in the blastopore of the cnidarian sea anemone *Nematostella vectensis* (*N. vectensis*) can be repressed by blocking gastrulation and can be partially re-established in response to uniaxial global deformation applied to early *N. vectensis* blastulae in a β-cat dependent way. Thus, the blastopore specification, which determines the ectoderm of the pharynx and has been proposed to separate the ectoderm from other tissue types (Servetnick et al., 2017; Steinmetz et al., 2017), was suggested to be mechanically induced by the gastrulation morphogenetic movement (Pukhlyakova et al., 2018).

The cnidarian *N. vectensis* and the bilaterians *Drosophila* and zebrafish diverged from the common ancestor of bilaterians and cnidarians at least 600 million years ago (Park et al., 2012; dos Reis et al., 2015). Thus, alternatively to biochemical signals, mechanical signals could be plausible initiators of the intracellular biochemical reactions that, in turn, resulted in the evolutionary emergence of EM specification and gastrulation in early Metazoa. Such mechanical signals could, in this case, have been conserved during evolution and in current species of cnidarians and bilaterians.

Here this hypothesis was evaluated in the marine mechanical, environmental context in which pre-metazoan multicellular colonies evolved, using both evolutionarily pivotal multicellular and colonial species: the metazoan cnidarian *N. vectensis* and the choanoflagellate *Choanoeca flexa* (*C. flexa*)*.* The common ancestor of these species with bilateria dates back 600–700 million years and more than 700 million years ago, respectively (Park et al., 2012; dos Reis et al., 2015). Soft waves were used to mimic the hydrodynamic mechanical stresses developed at the marine coastlines and applied to both species to test the sharing of mechanotransductive activation of i) the myosin-dependent morphogenetic movement of inversion of the geometric curvature of multicellular leaflets that initiates metazoan gastrulation and ii) Y654-βcat phosphorylation involved in EM specification in these evolutionarily distant species of Bilateria. The sharing of mechanical stimulation of EM morphogenesis and specification could allow the dating of the evolutionary emergence of these mechanical/molecular processes, possibly conserved relative to the last common ancestor of these two species with Bilateria.

## Materials and methods

### Nematostella vectensis

#### *N. vectensis* culture and spawning

*N. vectensis* sea anemones (WT, males and females, more than 1-year-old, accession number ABAV01000000) are kept in artificial seawater (ASW) at 18°C in the dark and fed every day with fresh *Artemia* nauplii. Females and males are kept separately in different glass bowls (ϕ15x6 cm), half-filled with ASW made by dissolving 10 g of Instant Ocean salt (Aquarium Systems) in 1 L of deionized water (1/3x SW). Approximately 30–40 individuals are cultivated in each bowl. Animals are moved to clean bowls with fresh 1/3x SW every 2 weeks. No further care is required during the weekend.

Adult animals were prepared for spawning every 3 weeks. Animals are fed with defrosted oysters 24–48 h before spawning day. The night before spawning, the animals/culture medium temperature was slightly increased (∼25°C) and exposed to light for 9 h. After this spawning stimulation, animals are moved to new bowls with fresh, cool (18°C) 1/3x SW awaiting the release of gametes. Sperms and eggs are collected separately. Oocytes are fertilized *in vitro* to synchronize development by dropping sperms into the egg beaker, which is then gently shaken for 15 min at room temperature. The fertilized eggs are kept in the dark at 18°C. In this condition, most embryos reach the blastula stage 18 h after fertilization. For each individual experiment, all embryos (controls and perturbed embryos) are taken from the same lay synchronized by common fertilization in the same solution before being separated, treated, and fixed in parallel at the same time. Thus, they are analyzed at the same stage.

#### Hydrodynamic stimulation experiment

A liquid silicone-based elastomer (Sylgard 184, Dow Corning) and a curing agent were mixed at a 10:1 ratio and then poured into ϕ 10 mm Falcon Petri dishes (4 g of mixture/dish). The dishes were left on a 37°C heater for 3 h. When the mixture was almost dried out but remained sticky, sand with an average grain size of 1 mm was added and spread out to fully cover the dish floor. Sanded dishes were rinsed thoroughly with MilliQ water and then 1/3x filtered sea water (FSW) before the experiment. These dishes can be reused.

The sand was glued to reflect the granularity of the seashore ground that kept sand on the ground under flow, whereas in the Petri dishes, the sand would have been removed by the flow leading to flat ground. The flow near the ground is probably slightly different at the 0.5–1 mm (size of sand grains) distance over the surface in the loose configuration compared to fixed sand but should remain on the same order of magnitude at the larger distance of 1.5–4 mm scale of the gel in which embryos are embedded. Indeed, because of the existence of a fixed sand surface below the loose one on the seashore, the flow velocities with sand glued should remain on the same order of magnitude compared to the seashore configuration. The granularity of the ground of a sea-shore natural environment should result in friction between the granular surface and the jelly because even the loose sand surface is necessarily a fixed granular sand surface inducing such friction.

At 16 h, embryos were transferred to sanded petri dish filled with 22 ml of 1/3x FSW. The dish was then put on shaker’s plateaus (Major Science MS-NOR-30), which rotated at 85 rounds per minute and reversed the direction of rotation every 2.5 s for 2 h, from 16 to 18 h. All experiments were performed at 18°C unless otherwise stated.

Jellied embryos were then de-jellied using an L-cysteine solution (0.4 g/10 ml of 1/3x FSW, pH adjusted to 7.4–7.6).

Stochastic stimulations were performed by applying the following stochastic rpm, duration (min), and period sequence:

**Table:** 

RPM	Duration (min)	Period (s)
58	5	5
81	27	2
76	16	9
98	22	6
76	29	5
55	28	2

Stochasticity was computer-generated randomly within the limit conditions of the instrumentation.

#### Morphologic measurements

The length *L* to width *W* ratio (ratio elliptic ratio) of hydrodynamically deformed embryos, and the invagination depth *d* divided by diameter *D* of the embryos were measured with ImageJ. The surface of apexes in the most curvature-inverted domain of the embryo, divided by their surface outside of the most curvature-inverted domain, was measured on nearly 20 cells each, on control and hydrodynamically stimulated embryos.

#### Oxygenation measurement

Dissolved oxygen was measured with a Hanna kit (Hanna instruments) using the HI764080 probe. The probe is calibrated at two points: i) 100% oxygen-saturated calibration when the probe is placed in the air near and above a water surface and ii) zero oxygen calibration when the probe’s membrane is dipped in Hanna HI7040 Zero oxygen solution. Once calibrated, the relative oxygen solubility is measured by directly submersing the probe tip into the solution. Relative oxygen solubility was measured just before and after 2 h of hydrodynamic stimulation in two duplicated independent experiments (N = 2).

#### Global compression experiment

Embryos were compressed between a coverslip and agar between 16 and 18 h (Brunet et al., 2013; Pukhlyakova et al., 2018).

#### Rapid camera deformation imaging

The videos were recorded using a uEye camera equipped with a Navitar 4X Zoom lens, providing a depth of field on the order of 100 µm. The movies were shot at 10 frames per second with a resolution of 1 μm per pixel. A Petri dish containing embryos was put on an LED light source attached to the shaker’s plateaus, and the whole system was placed under the camera. After 1 h of stimulation, the top-down 30 s videos were captured every 5 min in order not to overheat samples. The images extracted from the videos showed how embryos deformed in response to the stimulation. This deformation is measured by their length-to-width ratio.

#### Inhibitors

ML7 inhibitor (Sigma I2764) stock solution was prepared with 50% (v/v) ethanol at a concentration of 22 mM. ML7 treatment was carried out at 14 h and with a final concentration of 10 µM until the end of the experiment.

A commercial in-solution blebbistatin inhibitor was purchased from Sigma-Aldrich (2203389). Blebbistatin treatment was carried out at 14 h and at a final concentration of 50 µM until the end of the experiment.

The S381-0393 small molecule (https://www.chemdiv.com/search-element.php?q=S381-0393) has been selected *in silico* by ChemDiv upon request from *in silico*-designed inhibitors of βcat signaling targeting all of the βcat reactive and protein interacting site (https://www.chemdiv.com/catalog/focused-and-targeted-libraries/inhibitors-of-beta-catenin-signaling/?sphrase-id=40485). The S381-0393 was selected by ChemDiv as a specific inhibitor of Y654-βcat phosphorylation by mimicking best the α-helix subdomain of E-cadherin that includes I657, A656, and D665 and interacts with Y654 βcat to dock Y654-bcat and thus prevent its phosphorylation. It was validated experimentally by its effect on the inhibition of Y654-βcat phosphorylation in 21 h *N. vectensis* embryos in immunofluorescence at a final concentration of 100 µM (see Results section).

This compound was dissolved in DMSO because of good solubility. Only for experiments followed by an *in situ* hybridization labeling, because DMSO perturbed *fz10* expression, the compound was suspended at the same concentration in ASW (100 µM) instead.

Note that DMSO delayed embryonic development of 1 h, such that, in the presence of DMSO, embryos were fixed at the 19 h stage, equivalent to the 18 h untreated embryo stage.

#### Rhodamine phalloidin staining

Embryos were fixed in fresh 4% paraformaldehyde (PFA) supplemented with 0.2% glutaraldehyde in 1/3x FSW for 30 min at room temperature. Fixed embryos were thoroughly rinsed in PBS-Tween 1% (PTw) and then incubated in fetal bovine serum (FBS) at 10% (v/v). Finally, embryos were incubated in 300x diluted rhodamine phalloidin (Life Technologies, R415). Images were taken in black and white and colored in green for better visualization.

#### β-Catenin and p-Y654β-catenin staining

##### Immunohistochemistry

On the first day, fixed embryos were rinsed in PTw followed by FBS 10% (v/v) for 30 min. Then, embryos were incubated overnight at 4°C in the primary human antibody (anti-β-catenin produced in rabbit (Sigma, C2206) validated in *N. vectensis* in reference (Leclere et al., 2016) or human anti-p-Y654-β-catenin produced in mouse (CliniSciences, NB-22–0209-S and 1:250, Santa Cruz Biotechnology (1B11) Ref 57533)) for one night at 4°C.

The following day, embryos were thoroughly rinsed in PTw and again in FBS 10% for 30 min. After that, embryos were incubated in a secondary Alexa Fluor 488 conjugated antibody. Rhodamine phalloidin was also added in this step to the secondary antibody.

##### Western blot

*Nematostella* embryos (∼40–50 embryos per condition) were harvested at 18 h or 21 h of development and resuspended in 75 μL of ice-cold AT protein extraction buffer (20 mM HEPES pH 7.9, 1 mM EDTA, 1 mM EGTA, 20% glycerol, 1% Triton X-100, 20 mM NaF, 1 mM Na_4_P_2_O_7_.10H_2_O, 1 mM dithiothreitol, 1% phosphatase inhibitor cocktail, and 1% protease inhibitors). Embryos were pulverized in the AT buffer with a plastic pestle and sonicated twice for 10 s at setting 5 on a 550 sonic dismembrator. Afterward, NaCl was added to each lysate to get a final concentration of 150 mM NaCl. Samples were centrifuged at 16,000 *g* for 20 min at 4°C, and the supernatant was transferred to a fresh 1.5 ml microcentrifuge tube. 50 μL of ice-cold AT buffer was added to the remaining lysate pellet and resuspended by drawing it through a 27.5-gauge needle. Protein concentrations of the pellets were determined by Bradford. An equivalent quantity of protein (50 mg) was resolved by SDS–PAGE, transferred to a nitrocellulose membrane (Invitrogen), incubated o/n with TBS-Tween 5% milk supplemented with 1% bovine serum albumin blocking buffer, and hybridized with the appropriate antibodies for 4 h. The detection of proteins was realized using enhanced chemiluminescence (Amersham, ECL Prime Western Blotting Detection Reagent). The following antibodies were used: anti-p-Y654-β-catenin produced in mice [Santa Cruz Biotechnology (1B11) Ref 57533 1:1,000], anti-GAPDH produced in rabbit (Sigma Ref G9545 1:1,000) (HRP) conjugated secondary antibodies (Jackson ImmunoResearch, 1:1,000) in the same blot, with phosphoY654-βcat ∼70KD (https://www.clinisciences.com/autres-produits-186/anti-phospho-ctnnb1-tyr654-antibody-921000209.html) and GAPDH 37 KDa.

#### *In situ* hybridization of *fz10* gene

##### Fixation

We followed the fixation protocol described in Genikhovich&Technau DOI: 10.1101/pdb.prot5282. Briefly, embryos were rinsed in PTw, then rapidly pre-fixed in PFA 4% + glutaraldehyde 0.8% for 90 s, and finally fixed in PFA 4% for 2 h. Fixed embryos are then rinsed 3x in PTw, 2x in sterile water, 1x in pure methanol. All steps were done on ice. Embryos were stored at −20°C in pure methanol for at least one night.

##### Preparation of mRNA probe

pCS2 DNA recombinant plasmids were used as templates to amplify the cDNA inserts by PCR. The PCR products were used as templates to synthesize anti-sense RNA probes containing digoxygenin-11-UTP (Roche Biochemicals).

*In vitro* transcription was carried out using the T7 Ambion message machine kit (Ambion, United States) to synthesize anti-sense probes for *fz10*, *bmp-1 like.* After DNAse RNAse-free treatment, RNA was dissolved in RNAse-free water.

##### Procedure

We followed the *in situ* hybridization protocol optimized by E. Röttinger’s team (Rottinger et al., 2012). All duplicated experiments were realized and revealed with controls and perturbed conditions at the same time with the same mother solutions and timing conditions.

#### mRNA microinjection

##### Preparation of mRNA

The non-phosphorylatable dominant negative of Y654-βcat RNA, Y654F-βcat, was produced by mutating the nucleotide sequence of Y tyrosine “tac” in the F Phenylalanine sequence “ttc” on the Y641 of *N. vectensis* (http://nvertx.ircan.org/ER/ER_plotter/home) that, following a sequence alignment, was determined to correspond to the Y654 residue in mice (Roper et al., 2018). The sequence was cloned in pCS2+, and mRNA was produced with mMESSAGE mMACHINE^®^ Kit, High Yield Capped RNA Transcription Kit, SP6, Catalog Numbers AM1340.

*Stb-MO* and *stb* were designed (Kumburegama et al., 2011), with the following standard sequence as a Control-MO sequence: CCT​CTT​ACC​TCA​GTT​ACA​ATT​TAT​A.

##### Microinjection experiment

Embryos are maintained at 1/3x FSW at 16°C after being fertilized and dejellied to slow down their development. The injection must be done within the first 4 h after fertilization, before the first cleavage. For the injection experiment, embryos were deposited on a Falcon Petri dish (351007) along some parallel scratches, which served as landmarks with manipulation under a microscope. These dishes are recommended for good adhesion of embryos on the floor. We used home-made micro-needles fabricated by a capillary pulling machine. The FemtoJet microinjector enables one to adjust a constant, permanent pressure and injection pressure. In practice, these parameters are tuned so that the liquid volume at the needle end, when injected, makes a spot of diameter roughly 20% of the embryo diameter. Phymep micromanipulator SM3.25 was used.

Injected embryos are kept at 20°C. In this condition, embryos normally reach the blastula stage at 20 h. Before any further experiments, we select only successfully injected embryos by removing all broken or without detectable fluorescent marker embryos.

Hydrodynamic stimulation was thus applied between 18 and 20 h embryos for *stb-MO* experiments.

#### Imaging and analysis

##### Spinning and microscope

Fluorescent imaging and morphological observation were performed with a Yokogawa spinning dish confocal (CSU-X1) coupled to Olympus inverted microscope (IX70). The actin, nucleus, or β-cat of *N. vectensis* embryos in this study was an image extracted from a stack or MAX intensity projection of a whole stack obtained with ImageJ software.

Cell size analysis was performed on the max projection image of each stack. A 50 × 50 µm^2^ zone was chosen inside the invaginating domain to measure apex constriction in the gastrulating EM and outside, far from the invaginating area (when applicable, given the embryo orientation images) to avoid apex deformation by gastrulation. The average cell size was then determined by dividing the area by the number of cells within the zone.

The quantitative analysis of nuclear β-cat intensity was done in the invagination area (if applicable) or on domains of maximal nuclear β-cat intensity for non-invaginating embryos. The nuclear β-cat signal of each cell was normalized to the intensity of an equivalent area but outside of the nucleus in the cytoplasm of the same cell. For each embryo, this measurement was applied to about 10 cells randomly in the concerned domain.

Y654-β-cat phosphorylation quantitative analysis at the tissue scale was performed by ImageJ. Image stacks of the embryo were taken every two microns on half of the embryo. The total intensity/pixel of the embryo was measured and normalized to the average intensity of the external background after the summing of all stacks.

The intensity gradient was systematically measured based on the plan in the blastopore (invagination area) of maximal intensity, normalized to the intensity of the rest of the embryo. At 18 h of development, non-gastrulated embryos were systematically orientated along the direction of the maximum gradient for the measurement.

Y654-β-cat phosphorylation quantitative analysis at the cell scale was performed by an algorithm using the Phalloïdin junctional signal to discriminate between two different cells (see Code in Supplementary Material) after imaging stacks of images separated by 1 micron of each embryo along its three principal axes (Merle, 2018).

To precisely analyze large numbers of embryos at the cell scale, we created an algorithm using Python programming. 3D images (stack) were projected to get 2D images (sum of 5 pixels around the z maximum). We first applied an Otsu filter to discriminate the background signal from the embryo signal. Then, based on the actin signal, we realized skeletonization using the DisPerSE software (Sousbie, 2011). The skeleton was dilated up to 5 pixels to correspond to the width of the cell junctions. A watershed-based segmentation allowed us to create a cell catalog, reporting for each cell its apical area and its apical perimeter. Moreover, for each channel (actin and pY654-*β*cat signals), the catalog contains the mean intensities of the junctional and cytosolic cell signals, respectively. All these measures were normalized to the background mean intensity.

Hybridization images were taken with a CoolSNAP (HQ2) camera mounted on an upright widefield Leica microscope.

### Choanoeca flexa

#### Culture

The *C. flexa* strain containing the *Pseudomonas oceani* bacteria only (that lacks genes in the retinal photosensitive pathway biosynthesis) was obtained from Thibaut Brunet (UC-Berkeley) and cultured in 10 ml sea water medium in a sea salt 16.45 g/L culture flask, in the presence of rifampicin 20 μg/ml antibiotic to which *Pseudomonas oceani* is resistant, selecting this bacteria only into the culture (Brunet et al., 2019), with the difference that the antibiotic was directly diluted into the sea water medium without DMSO. Culture let to around 70 microns multicellular half-spheres. Adding a rice grain into the culture following reference (King et al., 2009) led, in addition, to numerous 500 to 1,000 microns multicellular sheets mixing half-spheres and almost complete spheres.

#### Hydrodynamic stimulation

Rice cultured *C. flexa* were hydrodynamically stimulated after 4–5 days of culture, for 2 min directly in three flasks, by the Major Science MS-NOR-30 shaker used for *Nematostella* hydrodynamic stimulation, with a speed of 105 rpm and a positive-to-negative cycling period of 2.5 s, leading to a flow on the order of 20 cm/s, after removing the rice grain.

#### ML7 treatment

10 ml of the sea water cultured *C. flexa* was implemented with 1–22.5 μL of a 22 mM concentration of ML7 suspended in 50% water and 50% ethanol for 30 min before hydrodynamic stimulation. Note that ML7 treatment did not perturb unstimulated *C. flexa* structures but systematically weakened hydrodynamically stimulated *C. flexa* structures, leading to smaller pieces of multicellular tissues after hydrodynamic stimulation.

#### Morphological observation

The *C. flexa* morphological phenotypes were observed in transmission with a DMIRB inverted microscope Leica recoded with a C4742-95 Hamamatsu camera. Phenotype counting was initiated 20 min after the end of the stimulation.

#### Fluorescent labeling and imaging

*C. flexa* were fixed following reference procedures described by Brunet et al. (2019) on FluoroDishes and mini glass slides pre-treated with a handheld Corona surface treater (Electro-Technic Products BD-20AC) and coated with poly-D-lysine (Sigma Aldrich P6407-5 MG). Immunofluorescence staining was performed using rabbit anti-myosin (1:10, M7648 Sigma Aldrich) primary antibody following reference^21^ and rhodamine phalloidin (1:100, Thermo Fisher Scientific). Secondary antibodies were used: anti-rabbit Alexa 488 (1:300, Invitrogen) and anti-mouse Alexa 647 (1:300, Invitrogen). Sections were cover-slipped with Prolong Gold Antifade (Thermo Fisher Scientific). Images were taken with a Nikon A1R 25HD confocal microscope at the Nikon Imaging Centre located at the Curie Institute. Images were processed using Fiji/ImageJ software.

Note that inverted and ML7-treated structures revealed much more fragility than non-inverted structures with regard to fixation, showing partly and rod-like fully inverted small patches of tissues for hydrodynamically stimulated conditions only and no multicellular structure at all in ML7-treated conditions.

Feeding efficiency essays on 200-micron fluorescent beads were performed following reference procedures as described by Brunet et al. (2019).

### Statistical tests

Statistical tests were the Fisher’s exact test (for the number of positive and negative embryos) and the non-parametric Mann–Whitney *U* test (for quantitative analysis associated with each embryo). In the specific case in which a bimodal distribution with an important difference between the two populations appears in the un-jelled stimulated condition, the five elements of the upper population were compared with five elements representative of the mean value of the downer population. The sample size was empirically increased until an initial tendency in a first experiment could be confirmed by a *p*-value <0.05 in a replicate. All experiments were replicated independently at least N = 2 times (biological replicates).

## Results

### Hydrodynamic mechanical stimulation triggers gastrulation in the cnidaria *Nematostella*

**FIGURE 1:**
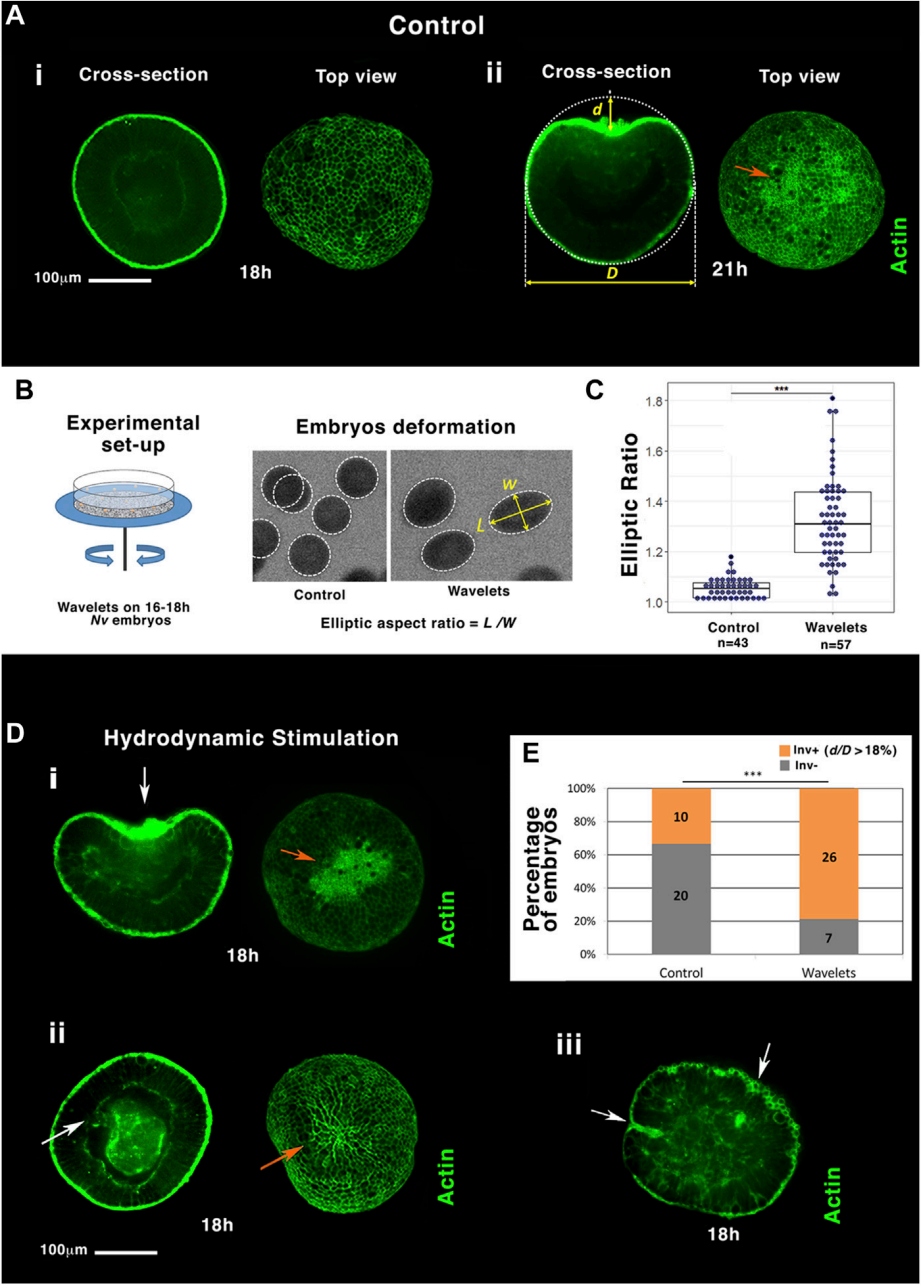
Hydrodynamic stimulation of *Nematostella* embryo gastrulation by the marine flow. **(A)** The most representative gastrulation state population of embryos at (i) 18 h and (ii) 21 h. **(B)** Setup: mimicking sea ocean stream by shaking a Petri dish with sand on its ground. Hydrodynamic deformation of jelled embryos with sand. **(C)** Quantification of the elliptic deformation of embryos. n_Control_ = 43 and n_Wavelet_ = 57, *p* = 3.10^–15^. Mann–Whitney statistical test. **(D)** i,ii, iii: embryos representative of the majority of individuals submitted to the flow (see text). White arrows: invaginations. Orange arrows: apex constriction. Scale bar is 100 μm. **(E)** Quantitative characterization of the percentage of gastrulating embryos (in orange), submitted to the flow compared to control (in grey), based on significant normalized invagination depth *d/D* measurement higher to 18% (see text), n_Control_ = 30 and n_Wavelet_ = 33, *p* = 7.10^–4^. Fisher statistical test. N = 2 biological replicates (embryo number inside columns).

In *N. vectensis*, EM development is initiated by its biochemical specification at the future oral pole of the embryo (Rottinger et al., 2012) that conditions its subsequent invagination (gastrulation) (Magie et al., 2007). As shown in Figure 1A, the *N. vectensis* embryo (of diameter D), still isotropic 18 h after fertilization (h), presents an invagination after 21 h of development at 18°C (of depth *d*). This invagination coincides with an apex contraction seen on actin-labeled gastrulating embryos (orange arrow), known to be a driving force of invagination initiation (Magie et al., 2007).

A Petri dish containing the embryos was shaken with a rotation amplitude of 2 cm radius, a speed of 85 rounds per minute, and a rotation orientation inversion every 2.5 s to test the effect of marine hydrodynamic mechanical stimulation in *N. vectensis* gastrulation. This corresponds to a flow with a velocity of ∼10 cm/s, consistent with marine streams, with a periodicity of ∼2 s, also consistent with the soft-ending wavelets on the sea shoreline environment (Figure 1B, Supplementary Info 1) (Prants et al., 2018). Embryos were first stimulated inside their deformable protective jelly, ∼1 cm in diameter (Rottinger et al., 2012). Sand (∼1 mm grains, Supplementary Figure 1A) has been glued on the Petri dish to approach non-smooth ground surface natural conditions, which should enhance the friction at the jelly surface on the ground (see section materials and methods). By taking advantage of the transparency of the jelly, embryos were observed by fast imaging and found to be deformed by the substrate rotation to adopt an ellipsoidal shape characterized by its elliptic ratio (Figure 1B, see section materials and methods). The mean elliptic deformation L/W (L is the major axis and W the minor axis) was nearly 5% under static conditions and increased to 30% under hydrodynamic flow (Figure 1C, Supplementary Figure 1B). Deformation was mostly elastic as embryos mostly returned to their original shapes after stimulation.

Blastulae were hydrodynamically stimulated for 2 h between 16 and 18h, in a stage normally known to be largely non-gastrulating. Embryos were then dejellied and fixed, and actin filament was labeled with phalloïdin at 18 h (3 h before the onset of gastrulation of un-perturbed embryos, Figure 1A). We observed that gastrulation was initiated at 18 h in 78% of the hydrodynamically stimulated embryos (Figures 1D,E), compared to the 33% of invagination observed in the static controls (Figures 1A,i, E-control). This represents 67% of the pool of non-invaginated embryos in static conditions. The hydrodynamically induced gastrulating embryos show an invagination with a normalized depth d/D greater than 18% (Figure 1E), a value from which apex contraction is observed in the invaginating tissue (Figure 1D, orange arrow, Supplementary Figure 1C, and Supplementary Info 2A).

In ∼ 60% of cases, gastrulating embryos showed anomalous shapes: ∼30% of embryos exhibited large invaginations enlarging their lateral size (first row of Figure 1D,i), and ∼30% of embryos showed tubular-like and multiple invaginations (last photo, second row of Figure 1D,ii,iii). Although most of the stimulated invaginations look anomalous (wider, tube-like, and multi-invagination sites), all embryos for which we let enough time to reach planula larva stage maturity were as viable as control embryos (data not shown). Oscillating soft waves on the shore cannot be perfectly tuned on a given period. Therefore, we introduced stochasticity in the stimulation of 68–99 rpm and 2–9 s, with stochastic durations from 5 to 29 min for 2 h (see section materials and methods). Stochastic stimulation led to the hydrodynamic mechanical induction of gastrulation at least as efficiently as the mono-period and rpm stimulation, indicating that gastrulation is robustly stimulated by stochastic hydrodynamic stimulation closer to the more natural marine environment (Supplementary Figure 1D). Dejellied embryos also showed a significant invagination response after hydrodynamic stimulation, although less sensitive (Supplementary Figure 1E,ii-and Supplementary Info 2B). Hence, hydrodynamic force stimulation directly applied to embryos initiates gastrulation as well.

Alternative to mechanical deformation, wavelet movements might also increase water oxygenation, thereby leading to a global acceleration of the overall embryonic development in *N. vectensis*. However, no significant increase in oxygenation after the 2 h of hydrodynamic stimulation was observed. Indeed, relative oxygen solubility in sea water of 79%+−1% was found before and after 2 h of hydrodynamic stimulation (N = 2, duplicated experiment; see section materials and methods), excluding any involvement of oxygenation in the hydrodynamic stimulation for 2 h of 18 h *N. vectensis* embryo gastrulation. Moreover, the expression level of the developmentally expressed zygotic patterning gene *bmp1-like*, known to be strongly upregulated between 18 and 21 h of development (Supplementary Figures 2A,B and Supplementary Info 2C) and NvERTx.4.130707 expression profile on NvERTx database (Amiel et al., 2017), was not upregulated in stimulation compared to control embryos (Supplementary Figures 2A,B). In addition, the size of the embryonic cell apex should decrease with the developmental stage due to asynchronous divisions between 18 and 21 h (Fritzenwanker et al., 2007). We indeed observed an apex size reduction of ∼15 μm^2^ in 21 h invaginating controls compared to 18 h in non-invaginating controls (Supplementary Figures 2C,D). In contrast, no significant decrease was observed between 18 h non-invaginating controls and 18 h invaginating stimulated embryos, confirming that stimulated invaginating embryos are 18 h embryos. These observations confirm the absence of acceleration of embryonic development under hydrodynamic stimulation. Furthermore, direct mechanical stimulation of embryos squeezed by ∼ 20% between agar and a coverslip for 16–18 h led to triggering invagination similar to hydrodynamically treated embryos, further confirming mechanical strain as inductive of the invagination trigger (Supplementary Figures 2E–G).

A quantified flow-meter hydrodynamic oscillating laminar flow of ∼10 cm/s using the same but standing still Petri dish (Supplementary Figure 3A) further confirmed the hydrodynamic stimulation of invaginations (Supplementary Figures 3B,C).

These data show that hydrodynamic mechanical strain characteristic of the sea shoreline soft waves can trigger the apical contraction-dependent multicellular sheet curvature inversion, initiating embryonic gastrulation in the *N. vectensis* cnidaria representative blastula embryos (see Supplementary Info 2D).

### Hydrodynamical stimulation of *Nematostella* embryo gastrulation is Myo-II-dependent

**FIGURE 2:**
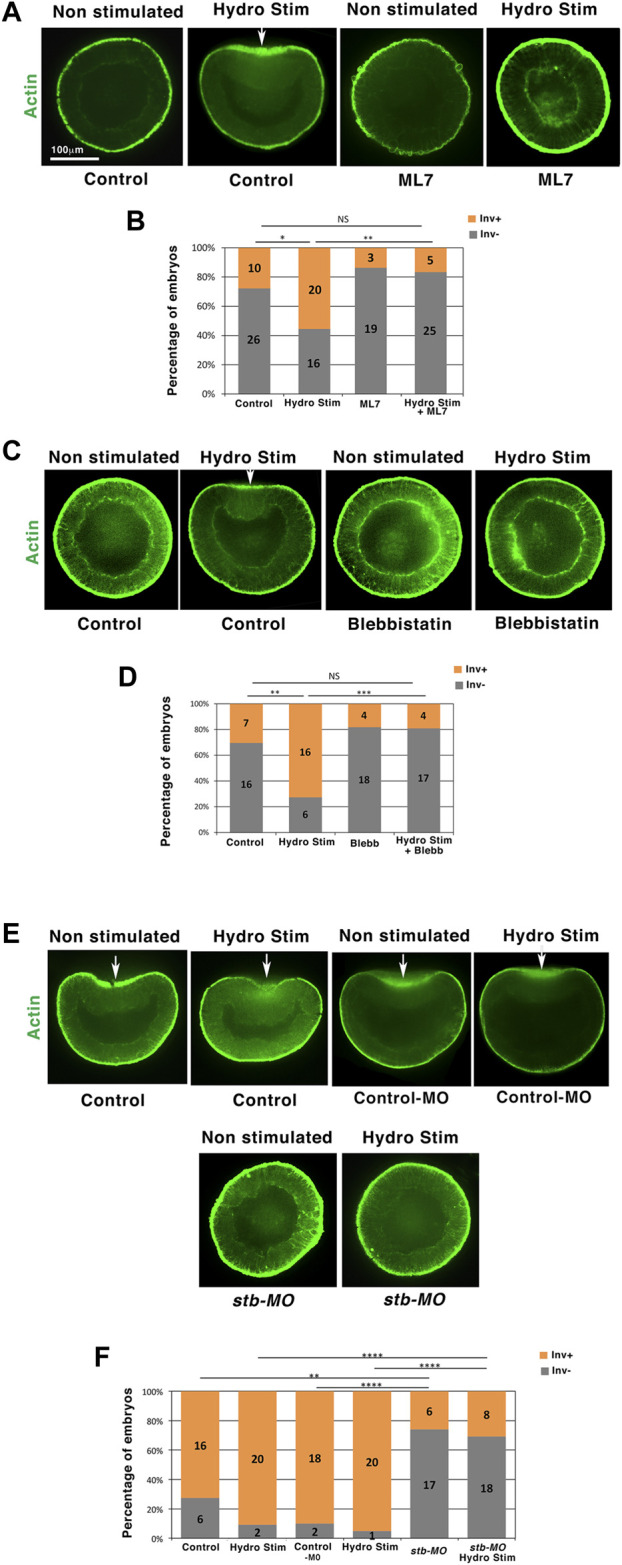
Hydrodynamic stimulation of *Nematostella* invaginations is Myo-II and *stb* dependent. **(A)** 18 h hydrodynamically stimulated *N. vectensis* embryos in the presence of the ML7 MLCK of Myo-II inhibitor. Control: ethanol, vehicle of ML7 (see methods). ML7-treated embryos show a slightly higher thickness than controls. **(B)** Quantitative analysis. n_HydroStim_ = 36 and n_HydroStim+ML7_ = 30 *p* = 1.9.10^–3^, n_HydroStim+ML7_ = 30 and n_Control_ = 36 *p* > 0.05. Statistical test: Fisher. **(C)** 18 h equivalent hydrodynamically stimulated *N. vectensis* embryos in the presence of the blebbistatin Myo-II inhibitor. Control: DMSO, vehicle of blebbistatin. **(D)** Quantitative analysis. n_HydroStim_ = 22 and n_HydroStim+Blebb_ = 21, *p* = 6.7.10^–4^, n_HydroStim+Blebb_ = 21 and n_Control_ = 23, *p* > 0.05. Statistical test: Fisher. **(E)** Hydrodynamically stimulated *Nematostella* 21 h embryos injected with the *stb-MO*. Control: water injected. Control-MO: injected with the MO control sequence (see section materials and methods). **(F)** Quantitative analysis. n_HydroStim_ = 22 and n_HydroStimStb-MO_ = 26, *p* = 2.7.10^–5^. N = 2 biological replicates. Fisher statistic test. White arrows: invaginations. The scale bar is 100 μm.

The apical contraction was observed in the invaginating tissue of all *N. vectensis* embryos initiating gastrulation with an inward curvature of more than 18% invagination depth (Figures 1A,D). Given the Myo-II dependence of apical constriction leading to gastrulation in many species (Young et al., 1991; Nance et al., 2003; Weiser et al., 2009), we tested in physiological conditions (jellied embryos) whether the Myo-II activity is required for mechanical, hydrodynamic stimulation of gastrulation. We used the ML7-specific inhibitor of Myo-II light chain kinase (Isemura et al., 1991) and the blebbistatin-specific Myo-II ATPase activity inhibitor (Kovacs et al., 2004) to block the Myo-II motor activity in *N. vectensis* embryos that remained in their natural jelly (Supplementary Info 3A). Following both treatments, we observed the inhibition of hydrodynamically induced apical constriction and gastrulation. At 18 h, the percentage of invaginating embryos subjected to flow was reduced by a factor of ∼3 to ∼4, which was similar to the non-stimulated control percentage (Figures 2A–D, Supplementary Info 3B).

To test whether the response of myosin II in *N. vectensis* is post-transcriptional like in *Drosophila* embryos (Pouille et al., 2009; Mitrossilis et al., 2017; Bailles et al., 2019), *N. vectensis* embryos were treated with actinomycin 2 h before hydrodynamic stimulation and during the 2 h of stimulation. Embryos did not survive drug treatment and disintegrated during treatment. Alternatively, Myo-II and phospho-Myo-II antibodies could also have been used to test for the activation of Myo-II *via* its phosphorylation and for the potential overexpression of Myo-II in response to the hydrodynamic treatment in *N. vectensis*. However, we did not find in the literature, or our experiments, any Myo-II and phospho-specific Myo-II antibodies that worked well in *N. vectensis.*

We observed hydrodynamically induced invaginations in multiple sites in a subset of embryos (∼25%, Figure 1D,iii). In addition, these invaginations remain grouped and polarized in one hemisphere of the embryo. This observation suggests the existence of a Myo-II mechanotransductive response that is genetically dependent on embryonic animal-vegetal pre-patterning. Interestingly, the expression of *strabismus* (*stb*), a gene upstream of Myo-II and mechanosensitively activable in vertebrates (Ossipova et al., 2015), is expressed at the animal pole (future oral pole) of *N. vectensis* embryos (Kumburegama et al., 2011). Required for gastrulation, it was proposed to be genetically upstream of Myo-II activation in *N. vectensis* (Kumburegama et al., 2011). We found that after injection of *stb* morpholinos (*stb-MO*), embryos failed to initiate gastrulation at 21 h, as well as following hydrodynamic stimulation, in line with the Myo-II-dependent hydrodynamic mechanotransductive stimulation of gastrulation (Figures 2E,F and Supplementary Info 4A,B). By labeling Stb in WT embryos, we found it to be expressed at one pole of the 18 h non-gastrulating embryos (Kumburegama et al., 2011), similar to the expression in the invaginating domain of hydrodynamically stimulated gastrulating 18 h embryos (Supplementary Figures 3D,E). This result confirms that Stb expression, which precedes hydrodynamic stimulation, is indeed required for mechanotransductive stimulation of Myo-II-dependent gastrulation in *Nv*. Interestingly, although Stb expression remained polarized after hydrodynamic stimulation, it was increased by a factor of nearly 2, suggesting Stb-dependent gastrulation response to hydrodynamic stimulation as re-enforced, or putatively triggered, by mechanically induced increased levels of Stb (Supplementary Figures 3D,E). In embryos, in which Myo-II is activated ectopically, gastrulation is inhibited due to the homogenous forces of gastrulation equilibrating and vanishing themselves all around the embryo (Morize et al., 1998). Consistently, we find the inhibition of hydrodynamic stimulation of 18 h embryo gastrulation after ectopic expression of Stb all around the embryo (Supplementary Figures 3D–G), further confirming that Stb is required for mechanical stimulation of Myo-II in *N. vectensis*. Interestingly, 30 min of hydrodynamic stimulation is not sufficient to trigger the inversion of curvature that initiates gastrulation, indicating that Stb-dependent mechanotransductive activation of Myo-II by hydrodynamic strains applied during 16–18 h is required for the mechanical initiation of gastrulation in 18 h embryos (Supplementary Figures 3H,I). These results suggest that Myo-II is mechanotransductively activated by hydrodynamic strains in an Stb-dependent process, thereby mechanically triggering the apical constriction that leads to curvature reversal and gastrulation initiation in 18 h *N. vectensis* embryos.

Together, these results indicate that the mechanical stimulation of Myo-II motor activity is involved in the process of hydrodynamic activation of apical contraction-dependent multicellular sheet curvature inversion that drives gastrulation by means of the flow (Supplementary Info 4C).

### Hydrodynamically induced gastrulation triggers *fz10* EM specification gene expression in *Nematostella* embryos

In today’s bilaterian embryos, early EM specification precedes, conditions, and regulates the first morphogenetic movements of embryogenesis (Tada et al., 2002; Roth, 2004; Solnica-Krezel and Sepich, 2012). In addition, the first morphogenetic movements of embryogenesis (invagination and convergent-extension in *Drosophila* and epiboly in zebrafish) subsequently participate in turn to the stimulation or induction of endomesodermal gene expression *via* the mechanotransductive activation of the β-cat pathway (Farge, 2003; Brunet et al., 2013). This activation is mediated by the mechanical activation and phosphorylation of the β-cat site Y654 (pY654-βcat). Thus, we tested in *N. vectensis* whether this property was already present in the common ancestor of bilateria and cnidaria.

**FIGURE 3:**
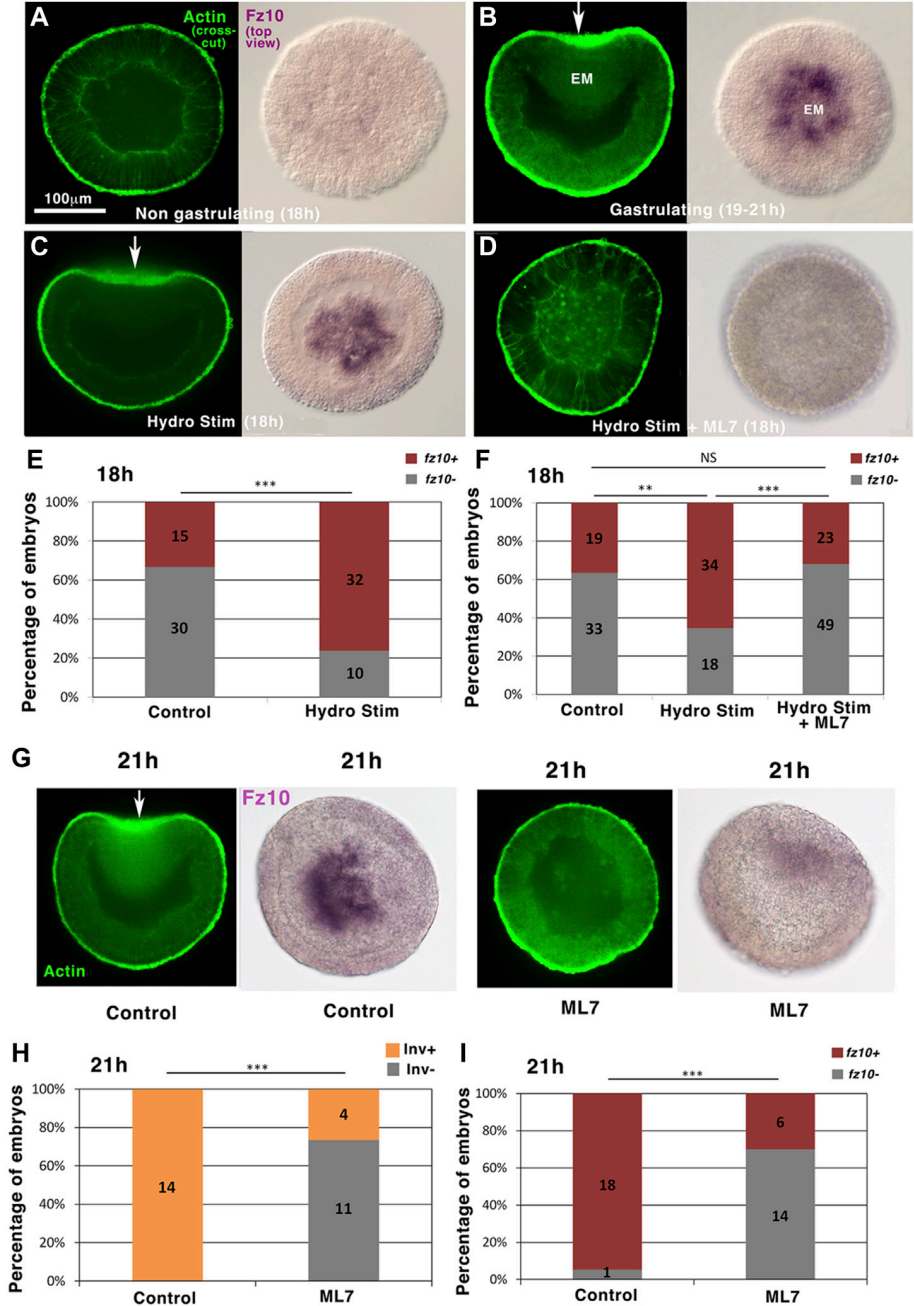
Hydrodynamically induced and endogenous gastrulation of *Nematostella* embryos stimulates the endomesodermal *fz10* gene expression downstream of mechanical stimuli in a Myo-II-dependent process. **(A)** *fz10* expression in 18 h non-invaginating embryos **(B)** and in the invaginating endomesoderm of 21 h embryos. **(C)** *fz10* expression in the invaginating tissue of 18 h hydrodynamically stimulated embryos **(D)** and in hydrodynamically stimulated non-gastrulating embryos treated with ML7. **(E,F)** Quantitative analysis. n_Control_ = 45 and n_HydroStim_ = 42, *p* = 9.10^–5^, and n_Control_ = 52 and n_HydroStim_ = 52, *p* = 5.7.10^–3^, n_HydroStim_ = 52 and n_HydroStim+ML7_ = 72, *p* = 2.7.10^–4^. Control of **(F)** ethanol, vehicle of ML7. Fisher statistic test. **(G)** *fz10* expression in non-gastrulating embryos ML7-treated 21 h embryos **(H,I)** and quantitative analysis. n_Control_ = 14 and n_ML7_ = 15, *p* = 5.10^–5^, n_Control_ = 19 and n_ML7_ = 20, *p* = 3.9.10^–5^. Control: ethanol, vehicle of ML7. Statistical test: Fisher. N = 2 biological replicates. The scale bar is 100 μm.

We first confirmed by *in situ* hybridization labeling that the spatiotemporal expression of the endomesodermal β-cat target gene *fz10* (Rottinger et al., 2012; Warner et al., 2018) happens at EM invagination in *N. vectensis* at 21 h gastrulation stage (Figures 3A,B and NvERTx.4.56484 expression dynamics on NvERTx database). Embryo top views show purple *fz10* labeling inside embryos into the gastrulating tissues only. To test whether *fz10* is induced at 21 h (early gastrulation stage), coincidentally or stimulated by gastrulation morphogenetic movements downstream of mechanical stimuli, we first triggered gastrulation at 18 h by hydrodynamically stimulating gastrulation from 16 to 18 h (Figure 1D). We found that hydrodynamically stimulated embryos show *fz10* expression at the gastrulation site at 18 h (Figures 3C,E). Consistently, the percentage of *fz10* positive individuals of both the control and hydrodynamic stimulated embryos is similar to that of gastrulating positive individuals (Figures 1E, 3E). In contrast, hydrodynamically stimulated embryos in which gastrulation was blocked by the Myo-II light chain kinase inhibitor ML7 were defective in *fz10* expression (Figures 3D,F). However, mechanical deformation application by direct uniaxial deformation on ML7 treated embryos (of necessarily higher intensity than hydrodynamic shear stress stimulation; see Supplementary Info 5) induces a rescue of *fz10* expression, showing that ML7 *per se* cannot inhibit *fz10* expression in the presence of a mechanical strain (Supplementary Figures 4A,B). In addition, a dose-response experiment using ML7 at lower (not affecting gastrulation) and higher (affecting gastrulation) concentrations revealed that *fz10* expression is inhibited only when gastrulation is blocked (Supplementary Figure 4C). This close correlation between the gastrulation rate and *fz10* expression further confirms that ML7 *per se* does not prevent *fz10* expression, but the inhibition of Myo-II-dependent gastrulation mechanical strains prevents *fz10* expression. Interestingly, the lack of *fz10* expression was also observed in hydrodynamically stimulated embryos in which gastrulation was genetically blocked by *stb-MO* injection (Supplementary Figures 5A,B and Supplementary Info 6). These results show that hydrodynamic forces *per se* (i.e., without gastrulation induced) could not stimulate *fz10* expression. Rather, these results indicate that *fz10* expression in the invaginating *N. vectensis* tissue is triggered downstream of mechanical stimuli by the Myo-II-dependent mechanical strains generated during gastrulation.

This is further supported by the fact that non-stimulated 21 h embryos that were previously treated with ML7 and that do not gastrulate did not show *fz10* expression as it should be in the case of non-treated gastrulating embryos (Figures 3G,H and Figures 3G,I). This observation, in turn, indicates that endogenous (non-mechanically induced) gastrulation in *N. vectensis* stimulates *fz10* expression in the EM, downstream of mechanical stimuli (Farge, 2003; Brunet et al., 2013).

These results indicate that the expression of the EM βcat-downstream target gene *fz10* is mechanically induced in response to hydrodynamically stimulated gastrulation initiation several hours earlier than the normal transduction (16–18 h) and in response to endogenous gastrulation initiation at 21 h in *N. vectensis* embryos.

### Hydrodynamically induced gastrulation triggers *fz10* EM specification gene expression in a Y654-βcat phosphorylation-dependent process in *Nematostella* embryos

**FIGURE 4:**
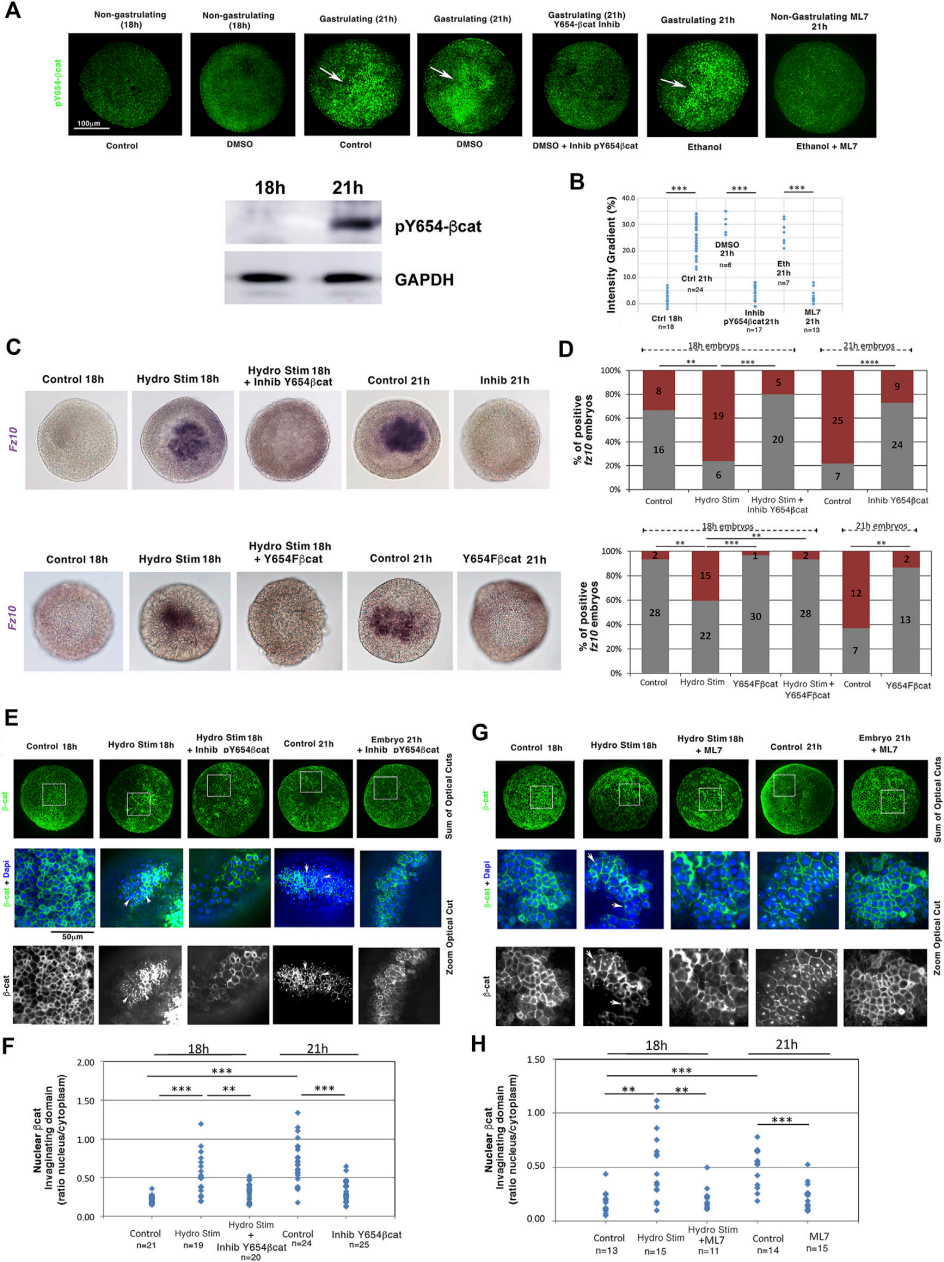
Mechanical induction of the endomesodermal *fz10* gene expression is initiated by the mechanical activation of Y654-β-cat phosphorylation. **(A)** Y654-β-cat phosphorylation in blastulae (18 h), gastrulae (21 h), and 21 h gastrulae embryos treated with either InhibY654-βcat, the inhibitor of Y654 phosphorylation, or ML7, the inhibitor of gastrulation. Western blot: Anti-pY654 β-catenin protein detection by western blotting of *N. vectensis* embryos at 18 h and 21 h of development. GAPDH was used as the loading reference. **(B)** Quantification of the gradient ratio (in percentage) of Y654-β-cat phosphorylation in invaginating compared to non-invaginating domains and from domains of maximal intensity compared to the other domains in non-gastrulating embryos of **(A)**. n_DMS021h_ = 6 and n_InhibY654bcat21h_ = 17, *p* = 2.3.10^–4^, n_Eth21h_ = 7 and n_ML721h_ = 13, *p* = 2.10^–4^. Mann–Whitney statistical test. **(C)** Expression of *fz10* in hydrodynamically stimulated 18 h embryos and in 21 h gastrulae embryos: (up) treated with InhibY654-βcat–control is DMSO, (down) and injected with the *N. vectensis* Y654F-βcat RNA dominant negative–control is H_2_0 injected. **(D)** Quantification of **(C)** (up) n_Control_ = 24 and n_HydroStim_ = 25, *p* = 4.10^–3^, and n_HydroStim_ = 25 and n_HydroStimInhibY654bcat_ = 25, *p* = 1.7.10^–4^ (down), n_Control_ = 30 and n_HydroStim_ = 37, *p* = 1.7.10^–3^, and n_HydroStim_ = 37 and n_HydroStimY654Fbcat_ = 31 1.7.10^–3^—Y654F-βcat 18h is not shown in **(C)**. **(E)** Nuclear β-cat translocation state in the non-invaginating blastulae 18 h tissue, in the 21 h invaginating tissue of the gastrulating embryos, and in the hydrodynamically stimulated 18 and 21 h invaginating tissues of gastrulating embryos treated with the Y654-β-cat phosphorylation inhibitor. Control and hydrodynamically stimulated: DMSO, vehicle of Inhib-Y654-β-cat. Full embryos: sum of the optical cross-section on the half sphere. Zoom: a given optical crosscut corresponding to the white square location shown on full embryos. In invaginating embryos, observation is made in invagination lateral domains when signal intensity is lost in its middle. **(F)** Quantification of **(E)** n_Control18h_ = 21, n_HydroStim18h_ = 19, *p* = 6.10^–6^, and n_Control21h_ = 24, *p* = 8.6.10^–8^. n_HydroStim18h_ = 19 and n_HydroStimInhibY654bcat18h_ = 20, *p* = 1.6.10^–3^, n_Control21h_ = 24 and n_InhibY654bcat21h_ = 25, *p* = 7.10^–6^. **(G)** Nuclear βcat translocation states in both non-gastrulating ML7-treated 18 h hydrodynamically stimulated and 21 h embryos. Control and hydrodynamically stimulated: ethanol, vehicle of ML7. **(H)** Quantification of **(G)**. n_Control18h_ = 13, n_HydroStim18h_ = 15, *p* = 1.710^–3^, and n_Control21h_ = 14 , *p* = 10^–4^, n_HydroStim18h_ = 15 and n_HydroStimML718h_ = 11, *p* = 6.9.10^–3^, n_Control21h_ = 14 and n_InhibML721h_ = 15, *p* = 2.10^–4^. Statistical tests. Fisher for embryo number counting and Mann–Whitney for quantitative data by embryos. N = 2 biological replicates. The scale bar is 100 μm.

We then checked the behavior of the mechanosensitive β-cat that targets *fz10.* Y654-βcat phosphorylation (pY654-βcat) leads to the release of β-cat from the junctions to the cytoplasm and nucleus, which triggers EM gene expression in bilaterian embryos during gastrulation and epiboly (Farge, 2003; Brunet et al., 2013), as well as in human embryonic stem cells (Muncie et al., 2020). Y654-βcat is highly conserved in all Metazoa, including *N. vectensis* (Roper et al., 2018)*.* Its phosphorylation was monitored in *N. vectensis* using antibodies against the phosphorylated form of Y654 in βcat using immunohistochemistry, as assessed in western blotting on *N. vectensis* (Figure 4A, Supplementary Info 7, Supplementary Figure 6A; see section methods). At 21 h, *N. vectensis* embryos exhibited a pY654-βcat signal in the invaginating EM (white arrows, not observed in 18 h non-gastrulating embryos). However, in ML7 treated 21 h embryos (i.e., 21 h non-gastrulating embryos), this signal was missing (Figures 4A,B, Supplementary Figures 6B,C, Supplementary Infos 8, 9). Before 18 h, hydrodynamically stimulated gastrulating embryos also showed pY654-βcat in the invagination (Supplementary Figure 6D, Supplementary Info 10). The oral-aboral signal gradient of pY654-βcat levels is in the order of 20% (Figures 4A,B), in line with the EM-ectoderm gradient of Y654-βcat level, mechanically phosphorylated in the invaginating and stretched EM of gastrulating *Drosophila* and epiboly zebrafish embryos, respectively (Brunet et al., 2013).

This indicates that the phosphorylation of the Y654-βcat site is mechanically induced by the driving forces of gastrulation.

Blocking phosphorylation of the βcat residue Y654 using the specific inhibitor S381-0393 (Y654-βcat Inhib) from 16 to 18 h and from 18 to 21 h, respectively (Figures 4A,B; see section materials and methods for validation of the inhibitor), led to defects in *fz10* expression in the invagination of both 18 h embryos in which gastrulation initiation was hydrodynamically stimulated and 21 h endogenously early-gastrulating embryos (Figures 4C,D, Supplementary Info 11A). This result was confirmed by injecting mRNA encoding a dominant negative version of *N. vectensis* βcat (Y645F) in which the tyrosine (Y) in residue 645 has been replaced by a phenylalanine (F) to prevent phosphorylation and thus activation (Figures 4C,D, Supplementary Figure 6A, Supplementary Info 11B). This shows that the mechanical activation of the EM gene *fz10* in the invagination by either hydrodynamically induced or endogenous morphogenetic movement of gastrulation initiation requires the phosphorylation of Y654-βcat, the latter of which is mechanically induced by gastrulation movements.

Consistently, β-cat translocation to the nucleus, unobservable in the 18 h non-gastrulating embryos, was detected in the invaginating domains of embryos that were hydrodynamically stimulated between 16 and 18 h and in early gastrulating embryos at 21 h (Figures 4E–H, Supplementary Info 11C). Moreover, blocking Y654-βcat phosphorylation with Y654-βcat Inhib (Figures 4A,B, Supplementary Figure 6B) prevented nuclear β-cat nuclear translocation in 18 h embryos in which gastrulation initiation was hydrodynamically stimulated from 16 to 18 h, as well as in early gastrulating embryos at 21 h (Figures 4E,F). In addition, in embryos that were hydrodynamically stimulated between 16 and 18 h and in non-stimulated 21 h embryos, both treated with the ML7 inhibitor of Myo-II light chain (i.e., non-gastrulating embryos), β-cat nuclear translocation was defective (Figures 4G,H).

These results indicate that in the cnidaria *N. vectensis*, the nuclear translocation of β-cat in the invagination and the expression of its EM target gene *fz10* depend on the mechanical stimulation of Y654-βcat phosphorylation triggered by the inititation of the morphogenetic movement of gastrulation that can be hydrodynamically induced several hours earlier than the normal transduction (18 h), and in response to endogenous gastrulation initiation, at 21 h.

### Hydrodynamic stimulation triggers curvature inversion in the choanoflagellate *C. flexa* multicellular sheet in a Myo-II-dependent process

To test whether hydrodynamically induced and Myo-II-dependent multicellular sheet curvature inversion could be found in an organism more evolutionary distant to bilateria than a cnidarian, whose common ancestor with bilateria is older than that of cnidaria and bilateria, we submitted the multicellular choanoflagellate *C. flexa*, a sister group to all Metazoa (Ruiz-Trillo and de Mendoza, 2020) to wavelet stimulation.

**FIGURE 5:**
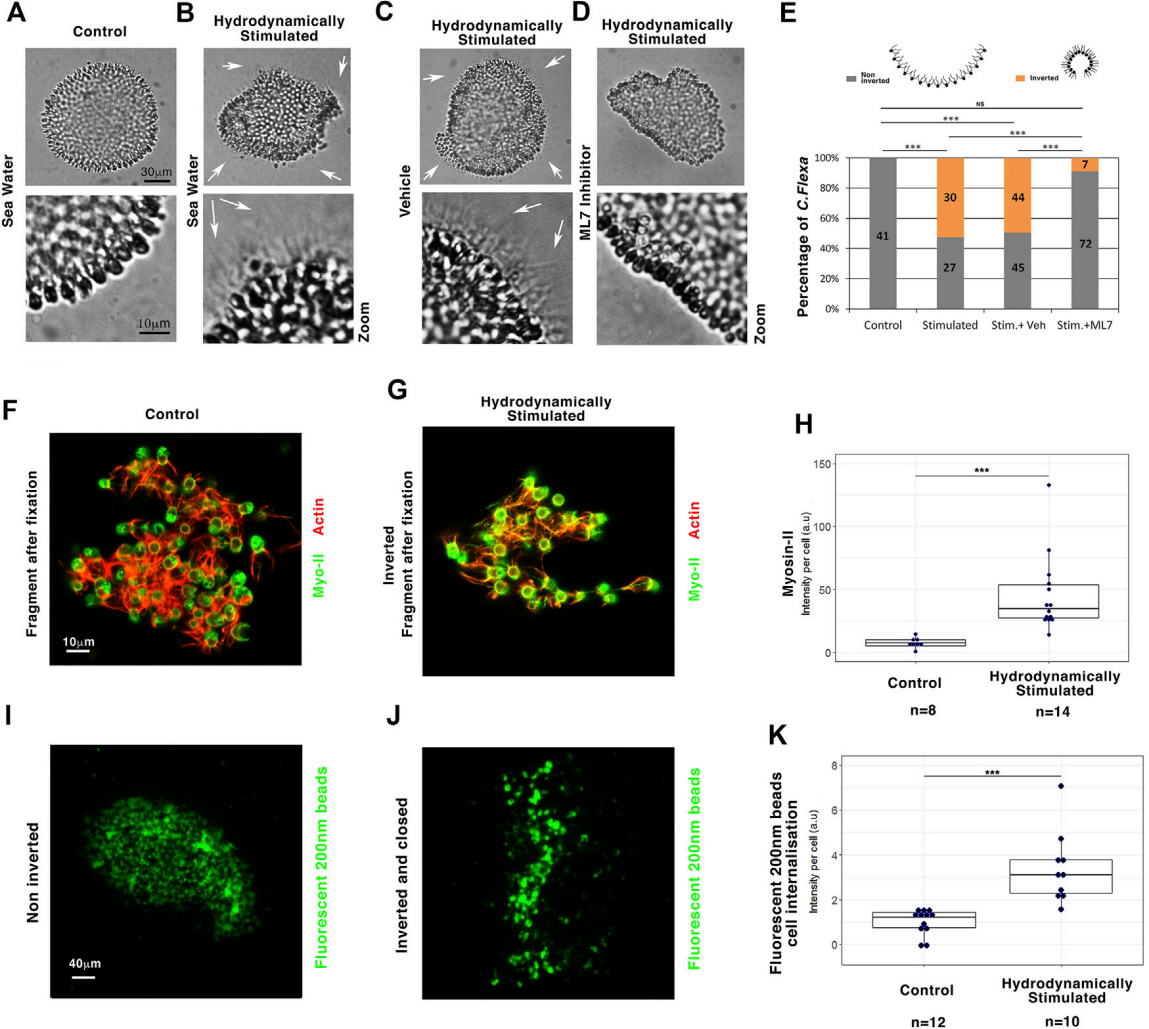
Myo-II-dependent hydrodynamic stimulation of multicellular sheet curvature inversion is conserved in *C. flexa* choanoflagellates. **(A)** Multicellular *C. flexa* choanoflagellate before hydrodynamic stimulation (no flagellate outside) **(B)** and after hydrodynamic stimulation (white arrows: flagellate outside). **(C)** Inversion status of hydrodynamically stimulated *C. flexa* treated with the ethanol vehicle of ML7 alone (white arrows: flagellate outside) **(D)** and with the Myo-II inhibitor ML7 (no flagellate outside). Zooms: with enhanced contrast to check for flagellate presence. **(E)** Quantitative analysis. n_Control_ = 41 and n_Stimulated_ = 57, *p* = 10^–9^, n_Stim+veh_ = 99 and n_Stim+ML7_ = 79, *p* = 4.8.10^–9^. Scheme from Brunet et al. (2019). **(F)** Myo-II and actin in *C. flexa* before hydrodynamic stimulation **(G)** and after hydrodynamic stimulation. **(H)** Quantitative analyses of Myo-II expression per cell 20–30 min after hydrodynamic stimulation initiation. Immunofluorescence representative of *n* = 8 controls and *n* = 14 inverted structures of the hydrodynamically stimulated *C. flexa*. n_Control_ = 8 and n_Stim_ = 14, *p* = 1.25.10^–5^. **(I)** 200 nm fluorescent beads internalization in cells in non-inverted **(J)** and inverted *C. flexa* after hydrodynamic stimulation. **(K)** Quantitative analyses of particle internalization per cell 60 min after hydrodynamic stimulation initiation. Immunofluorescence representative of *n* = 12 non-inverted and *n* = 10 fully inverted structures of the hydrodynamically stimulated *C. flexa*. n_Control_ = 12 and n_Stim_ = 10, *p* = 9.9.10^–5^. N = 2 biological replicates. Statistical tests are Fisher for histograms and Mann–Whitney for quantitative analysis.

*C. flexa* is an open curved multicellular sheet whose cohesion is ensured by collar microvilli interactions, those of flagella inward of the curved surface (flagella-in). Its curvature can inverse through apex contraction in response to light-to-dark transition through a Myo-II-dependent multicellular sheet contraction process, characterized by flagellates facing outward instead of inwards (see section materials and methods) (Brunet et al., 2019). Strikingly, under conditions in which retinal-dependent light sensitivity was removed in the absence of retinal-producing bacteria [see section materials and methods (Brunet et al., 2019)] and under light at a constant intensity, hydrodynamic wavelet stimulation of *C. flexa* colonies at 105 rpm and a rotation orientation inversion every 2.5 s triggered an inversion of *C. flexa* colony sheets curvature with outward flagellates (flagella-out) (Figures 5A,B,E, Supplementary Info 12).

Hydrodynamically induced inversion also triggered a transition to a swimming state, possibly due to the freed flagellates because of curvature inversion, similar to light-induced inversion of *C. flexa* (Brunet et al., 2019) (Supplementary Movies S1, S2, S3). Inhibition of Myo-II activity by ML7 treatment led to the inhibition of hydrodynamically induced inversion. Thus, Myo-II is necessary for the light modulation-independent hydrodynamically induced inversion of the multicellular form of *C. flexa* (Figures 5C–E and Supplementary Movies S4, S5). Interestingly, hydrodynamically stimulated inversion was accompanied by an intracellular increase of Myo-II expression of a factor of 4, 30 min after stimulation, possibly involved in the Myo-II-dependent mechanical induction of inversion (Figures 5F–H, Supplementary Info 13A).

We then investigated the efficiency of *C. flexa* feeding in response to inversion using fluorescent beads of 200 nm in diameter (i.e., the size of their bacteria prey) (Brunet et al., 2019). Quantification of the data revealed an increase in the feeding efficiency by a factor of nearly 2 in closed inverted (flagellate-out) *C. flexa*, compared to agitated non-inverted (flagella-in) (Figures 5I–K and Supplementary Info 13B). These results suggest that mechanotransductively induced inversion/tissue closing increases feeding efficiency, promoted by the trapping of suspended nutrients (Supplementary Info 13C,D).

Our data indicate that marine hydrodynamic mechanical stimulation triggers the curvature inversion morphogenetic movement of the choanoflagellate *C. flexa* multicellular sheet in a Myo-II-dependent process, leading to an inversion that favors the closure of the *C. flexa* and consequently their feeding capacity.

Due to the lack of β-cat in the choanoflagellate genome repertoire (Fernandez-Sanchez et al., 2015), which we confirmed in *C. flexa* associated with the lack of *fz10* (see Supplementary Info 14), no mechanical induction of β-cat activation and *fz10* expression by inversion could be tested in *C. flexa*.

## Discussion

In addition to multicellularity and reproduction by the egg–sperm fusion, the evolutionary emergence of the first Metazoa organisms is considered to have been conditioned by the formation of a first organ: the primitive gut (Haeckel, 1874; Brunet and King, 2017; Cavalier-Smith, 2017; Newman, 2020; Ruiz-Trillo and de Mendoza, 2020). This primitive gut is thought to consist of biochemically specified EM, fated to invaginate by the inversion of the geometric curvature of the multicellular tissue (gastrulation) from primitive multicellular hollow spheres (blastulae) (Arendt, 2004).

Due to the absence of conserved biochemical signals upstream of both EM biochemical specification (Roth, 2004; Kusserow et al., 2005; Hagos and Dougan, 2007) and biomechanical initiation of gastrulation (Sweeton et al., 1991; Kumburegama et al., 2011; Evren et al., 2014) in present-day Metazoa embryos, the existence of a common evolutionary origin responsible for the development of the primitive digestive organ in Metazoa remains unknown.

Interestingly, mechanical signals have been found to play an increasingly important role in early embryonic development (Brouzes and Farge, 2004; Wozniak and Chen, 2009). Indeed, the activation of Myo-II by internal mechanical strains has been shown to cause invaginations of the mesoderm and endoderm during gastrulation in *Drosophila* embryos (Mitrossilis et al., 2017; Bailles et al., 2019)*.* In addition, mechanical stresses caused by early internal morphogenetic movements during embryogenesis were found to stimulate or induce endodermal and mesodermal gene expression in bilaterian *Drosophila* and zebrafish early embryos *via* the mechanical activation of the β-cat pathway by Y654-βcat phosphorylation (Desprat et al., 2008; Brunet et al., 2013). Therefore, we tested the plausibility of mechanical cues as tangible candidates involved in intracellular biochemical reactions, which led to the evolutionary emergence of EM specification and invagination. This theory was tested on two evolutionarily distant organisms spanning at least 700 million years of evolution in the marine hydrodynamic mechanical context of their common ancestors—the cnidarian *metazoan N. vectensis* and the multicellular choanoflagellate *C. flexa*—considered to be the closest living relative of the metazoans.

### Myosin-dependent marine environmental mechano-biochemical stimulation that leads to contraction and inversion of pluricellular sheets is a shared property of the evolutionarily distant cnidarian and choanoflagellate species, whose common ancestor dates back at least to the last common ancestor of Metazoa

The results showed that reminiscent of bilateria (Fernandez-Gonzalez et al., 2009; Pouille et al., 2009; Mitrossilis et al., 2017; Bailles et al., 2019), the mechanical stimulation of Myo-II-dependent multicellular sheet inversion, which initiates gastrulation in closed blastulae, is shared by cnidaria embryos (*N. vectensis*) and multicellular choanoflagellates (*C. flexa*).

This indicates that Myo-II-induced mechanical contraction, leading to the inversion of the curvature of multicellular sheets in response to environmental, mechanical stresses, such as sea flow and waves on the shoreline, possibly dates back at least to the last common pre-Metazoa ancestor of the Metazoa and choanoflagellates, over 700 million years ago. Alternatively, the mechanical induction of Myo-II-dependent contraction leading to the inversion of the curvature of multicellular leaflets arose and was selected independently in these representatives of the bilaterian, cnidarian, and choanoflagellate superphyla, which have been tested, in the process of convergent-evolution. However, hydrodynamic mechanical strains do not represent the environment of gastrulating embryos of Bilateria insects, such as *Drosophila* embryos. Furthermore, environmental mechanical strains are not necessary for normal endogenous gastrulation of bilateria *Drosophila* and cnidaria *N. vectensis* embryos. This may therefore make it less likely that the mechanical stimulation of Myo-II-dependent multicellular sheet contraction leading to inversion is the result of a convergent-evolutionary process in three evolutionarily distant species (including the choanoflagellate *C. flexa*) compared to a single evolutionary-innovation process inherited at least from the earliest pre-Metazoa (see Supplementary Info 15).

**FIGURE 6:**
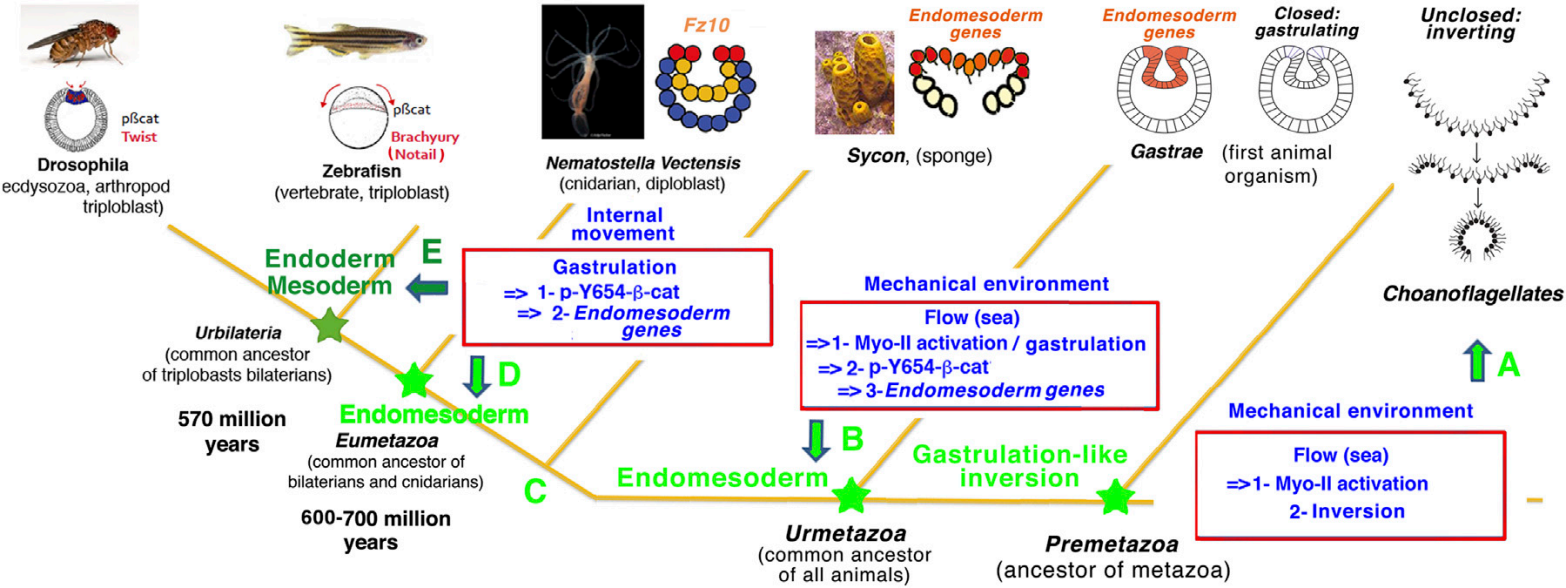
Mechanotransductive origins of first Metazoa animal emergence evolutionary: a possible scenario. **(A)** Results: *C. flexa* unclosed multicellular choanoflagellate inversion [scheme from Brunet et al. (2019)] are mechanotransductively stimulated by hydrodynamic flow mimicking soft waves on the sea shore in a Myo-II-dependent mechanotransductive process (Figure 5), like *N. vectensis* cnidaria gastrulation (Figure 1) and the mechanotransductive activation of *Drosophila* bilateria gastrulation (Mitrossilis et al., 2017). Evolutionary possible scenario: Myo-II mechanotransductive induction of constriction leading to multicellular inversion/gastrulation as possibly conserved along Metazoa evolution, from the hydrodynamically induced inversion of pre-Metazoa unclosed multicellular choanoflagellate. **(B)** Results: environmental hydrodynamic sea water hydrodynamic forces lead to mechanotransductive induction of gastrulation, with gastrulation strains mechanotransductively stimulating endomesoderm *fz10* gene expression in the gastrulating tissue of the cnidaria *N. vectensis* in a Y654-β-cat phosphorylation activation mechanotransductive process conserved with bilateria (Brunet et al., 2013) (Figures 3, 4). Evolutionary possible scenario: pre-Metazoa inversion that led to hydrodynamic induction of gastrulation after closing multicellular choanoflagellates, with gastrulation strains mechanotransductively leading to endomesoderm gene expression in gastrulating tissue after evolutionary emergence of β-cat mechanosensitivity, as possible conditions of the ur-Metazoa emergence. **(C,D)** Results: internal morphogenetic movements of gastrulation lead to endomesodermal *fz10* gene expression in the *N. vectensis* cnidaria embryo in a Y654-β-cat phosphorylation-dependent mechanotransductive process (Figures 3, 4). This is similar to the expression of Twist and *brachyury* endoderm and mesoderm genes in the early *Drosophila* and zebrafish bilateria embryos (Brunet et al., 2013). Evolutionary possible scenario: genetically regulated internal morphogenetic movements (gastrulation) possibly replaced external environmental (i.e., seawater) mechanical strains to stimulate gastrulation and endomesoderm specification in the Eumetazoan common ancestor of cnidaria and bilateria, thereby having initiated autonomous Metazoa embryogenesis. **(E)** The latter property has been possibly conserved in mesoderm and endoderm specification after endoderm/mesoderm divergence at the origin of the first bilateria emergence, as found in early *Drosophila* and zebrafish embryos that directly diverged from the common ancestors of bilateria (Brunet et al., 2013).

Therefore, the present work suggests that Myo-II-dependent mechanosensitive constriction by marine environmental strains in multicellular sheets may have been at the evolutionary origin of inversion in multicellular pre-Metazoa. It could have been ancestral to gastrulation by prefiguring the tissue inversion that initiated and triggered gastrulation in early Metazoa (Figures 6A,B).

In *N. vectensis*, a possibility could have been that Myo-II would already be activated in Stb-expressing domains in 16–18 h embryos independently of hydrodynamic stimulation, but not enough to cause curvature inversion leading to gastrulation initiation. In this case, hydrodynamic forces could have added to pre-existing Myo-II-dependent forces that pre-stressed the Stb-expressing domain, ultimately resulting in curvature reversal and gastrulation initiation.

Notably, if the tissue has already been pre-stressed by pre-existing Myo-II activity, then as soon as curvature reversal is initiated by incidental external forces (in non-hydrodynamically stimulated control embryos) or hydrodynamically, one should observe apical constriction initiation due to Myo-dependent internal forces that participate with external forces in curvature reversal initiation. This should start at d/D > 0 in Supplementary Figure 1C and include the regime 0 < d/D < 0.18, in which constriction was systematically evaluated in the maximal inward curvature domain of the embryo. This is not the case in our study, as constriction is significantly observed in embryos only from and above the d/D = 0.18 state.

Moreover, if the curvature reversal leading to gastrulation initiation was purely mechanically induced by hydrodynamic forces in a pre-stressed Myo-II-activated tissue, one would expect an almost immediate hydrodynamic stimulation of curvature reversal dynamics of the order of the minute characteristic time scale, which is a known characteristic of the viscoelastic mechanical properties of embryonic tissues (Desprat et al., 2008; Doubrovinski et al., 2017). This has to be compared to the 2 h (16h–18 h) stimulation required to initiate gastrulation. Indeed, we found that 30 min of hydrodynamic stimulation is not sufficient to trigger the inversion of curvature that initiates gastrulation (Supplementary Figures 3H,I), which would be largely sufficient in the case of a purely mechanical response of the tissue pre-stressed by an already activated Myo-II that would take on the order of a few minutes only.

Therefore, Myo-II is not activated in 18 h non-gastrulating embryos. This shows that hydrodynamic mechanical stresses do not synergize with the already activated Myo-II to stimulate gastrulation at 18 h and further suggests that a gradual and probably cumulative process of mechanotransductive activation of Myo-II by hydrodynamic strains applied from 16 to 18 h is required for the mechanical initiation of gastrulation in 18 h embryos.

Along with the present 105 rpm marine-like stimulation submitted to *C. flexa* ((Nguyen et al., 2020), original BioRxiv pre-print version of the present article), 5 s short 600 rpm vortexing also led to *C. flexa* contraction (Reyes-Rivera et al., 2022). Such vortexing would correspond to a non-physiological regime of 10 waves per second. We find Myo-II mechanical induction of *C. flexa* inversion in response to a nearly six-time lower frequency, more relevant to physiological marine wave frequencies. We also find that the mechanical stimulation of Myo-II-dependent *C. flexa* inversion leads to an increase in choanoflagellate feeding efficiency (Figures 5I–K). With *C. flexa*, in the marine environment in which multicellular pre-Metazoa evolved, hydrodynamically induced inversion may have increased feeding efficiency by trapping nutrient bacteria or nutrients suspended in the water in the presence of shoreline or seabed flow.

Therefore, this process may have been selected as a favorable behavioral feeding reflex response to marine mechanical strains (Farge, 2003; Pouille et al., 2009).

### The βcat-dependent property of mechano-biochemical induction of EM gene expression by tissue deformation during gastrulation dating back at least to the last common ancestor of cnidaria and bilaterians

Our observations further show the mechanical induction of the EM *fz10* gene expression through phosphorylation of Y654-βcat by the morphogenetic movement of gastrulation initiation in cnidaria *N. vectensis* embryos (Figure 6D).

In *N. vectensis* embryos, the timing and causality of this second mechanotransductive process with regard to Myo-II mechanical activation are characterized by the fact that the inhibitor of Y654-βcat phosphorylation or the dominant-negative Y654-βcat does not prevent hydrodynamic stimulation of gastrulation, indicating that Y654-βcat phosphorylation is not required for hydrodynamic stimulation of Myo-II. Indeed, gastrulation is observed on (21 h) Y654-βcat Inhib (Figure 4A) and (21 h) Y654F RNA injected (Supplementary Figure 6A), as well as Hydro Stim 18 h Inhib pY654βcat (Figure 4E) embryos. In contrast, Myo-II inhibitors that inhibit hydrodynamic stimulation of gastrulation also inhibit hydrodynamic stimulation of the EM *fz10* gene expression downstream of Y654-βcat phosphorylation (Figures 3C,D,F, 4C,D). Herein, mechanical stresses associated with gastrulation mechanotransductively activate *fz10* EM gene expression rather than *via* biochemical activation of Myo-II. This is indicated by the fact that *fz10* expression can be rescued by uniaxial mechanical deformation in ML7-treated embryos in which the Myo-II activity is inhibited (Supplementary Figures 4A,B).

Therefore, there are two waves of mechanotransduction: the first is the mechanical activation of Myo-II in response to environmental hydrodynamic stresses, which leads to gastrulation, and the second consists of the mechanical activation of Y654-βcat phosphorylation by the morphogenetic movement of gastrulation initiation, which participates in EM specification *via* the induction of *fz10* expression.

This mechanotransductive cascade leading to the induction of EM biomechanical morphogenesis (gastrulation) and participating in its biochemical specification (*fz10* expression) is identical to the mechanotransductive cascade observed in the morphogenesis and participation in the specification of the mesoderm of *Drosophila* embryos during gastrulation (Brunet et al., 2013). In addition, the first morphogenetic movement of zebrafish embryos is also involved in mesoderm specification *via* phosphorylation of Y654-βcat in margin cells specifically stretched by epiboly (Brunet et al., 2013).

Therefore, the mechanism of Y654-βcat phosphorylation by early morphogenetic movements initiation during embryogenesis is shared between cnidaria (*N. vectensis*) and bilaterians [zebrafish and *Drosophila* (Brunet et al., 2013)]. This strongly suggests that the mechanical induction of EM gene expression by the βcat-dependent morphogenetic movements of gastrulation dates back at least to the last common ancestor of bilateria and cnidaria, 600–700 million years ago. Additionally, an underlying alternative process of convergent-evolution that led to mechanical activation of the β-cat pathway through Y654-βcat phosphorylation in representatives of bilateria and cnidaria independently cannot be entirely excluded. However, insofar as a β-cat comprising a Y654 site capable of interacting with E-cadherin arose concomitantly with E-cadherin that has trans-cellular adhesive properties [causally to the multicellularity in the evolutionary emergence of early Metazoa (Fernandez-Sanchez et al., 2015)], multicellular tissues may not have escaped to a mechanically induced opening of Y654-βcat under stress leading to its phosphorylation from the earliest to the most recent Metazoa (see Supplementary Info 16).

Given the lack of conserved biochemical signals involved in EM specification in present-day embryonic species (Roth, 2004; Kusserow et al., 2005; Hagos and Dougan, 2007), this suggests that βcat-dependent mechanosensitive EM gene expression may have been involved in the evolutionary emergence of EM specification in early pre-Metazoa. During evolution, this mechanosensitive and βcat-dependent mechanism responding to first morphogenetic movements at the early gastrulation stage may have been subsequently co-opted by both endoderm and mesoderm in bilateria embryos (Desprat et al., 2008; Brunet et al., 2013; Muncie et al., 2020) (Figures 6B–E, Supplementary Info 17).

Intriguingly, βcat-dependent activation of the *brachyury* EM gene has also been proposed to be activated at the time of inversion in sponge embryos (Figure 6C) (Leininger et al., 2014). Due to the absence of β-catenin–E-cadherin junctional complexes in choanoflagellates (Fairclough et al., 2013; Fernandez-Sanchez et al., 2015), this mechanism is expected to be absent in response to the reversal of curvature in *C. flexa* (Figure 6A), suggesting that the emergence of the junctional β-cat containing the major Y654 site of β-cat interaction with E-cadherins and adhesive cadherins capable of interacting with β-cat in the multicellular tissues of pre-Metazoa may have been one of the conditions required for the emergence of gastrulation-induced EM gene expression in early Metazoa (ur-Metazoa, Figure 6B). This conserved process could have subsequently been consolidated and diversified throughout evolution by the establishment of a patterned and biochemically regulated expression of EM genes (e.g., downstream of Wnts, Nodal, or Dorsal as observed today in a non-conserved species-dependent manner). Moreover, EM specification would have pre-patterned the domain of competence of mechanical or biochemical Myo-II activation (e.g., *via* Stb or Fog, as observed today in a non-conserved species-dependent manner), required to enable local invagination in a closed epithelium. In this way, the conserved mechanical cues observed here to be involved in EM specification and morphogenesis add to patterned EM specification pre-existing gene regulatory networks in early embryos of current species.

## Conclusion

The observations presented in this study suggest that tissue invagination and EM specification, which jointly define primitive gut formation in early Metazoa, may have been initiated by mechanotransduction as a favorable primitive sensory and behavioral feeding response of early multicellular pre-Metazoa to the hydrodynamic mechanical stress of the marine environment (Farge, 2003; Farge, 2013). Alternative to a convergent-evolution process, the conservation of the lineage found here from bilateria to cnidaria for the mechanical activation of Myo-II and β-cat and to multicellular choanoflagellates for the mechanical activation of Myo-II lends credibility to mechanotransductive processes at the evolutionary origin of the primitive emergence of a gut in early pre-Metazoa and multicellular Metazoa.

## Data Availability

The raw data supporting the conclusion of this article will be made available by the authors, without undue reservation.

## Supplementary material

Supplementary Movie S1 Unstimulated non-swimming spherical multicellular choanoflagellates still opened at one pole. Changes in the focal plane were induced in the movie to ensure that flagellates were not observed outside the tissue.

Supplementary Movie S2 Stimulated swimming inverted multicellular choanoflagellates with a point of attachment to the substrate maintaining it in the same optical area.

Supplementary Movie S3 Stimulated swimming inverted multicellular choanoflagellates free and, in this case, larger and laterally opened. To follow it up, the observation area has to be changed several times.

Supplementary Movie S4 Stimulated swimming inverted multicellular Choanoflagellates in the presence of the ethanol vehicle of ML7 alone.

Supplementary Movie S5 Stimulated non-swimming and non-inverted multicellular choanoflagellates in the presence of ML7. Changes in the focal plane were induced in the movie to ensure that flagellates were not observed outside the tissue.
