# Supplementary material for: Mechano-biochemical marine stimulation of inversion, gastrulation, and endomesoderm specification in multicellular Eukaryota
Source: Front Cell Dev Biol. 2022 Dec 1;10:992371. doi: 10.3389/fcell.2022.992371 (PMC9754125; doi:10.3389/fcell.2022.992371)
Supplement: Supplementary file 3 [file DataSheet1.pdf]

Supplementary Figures

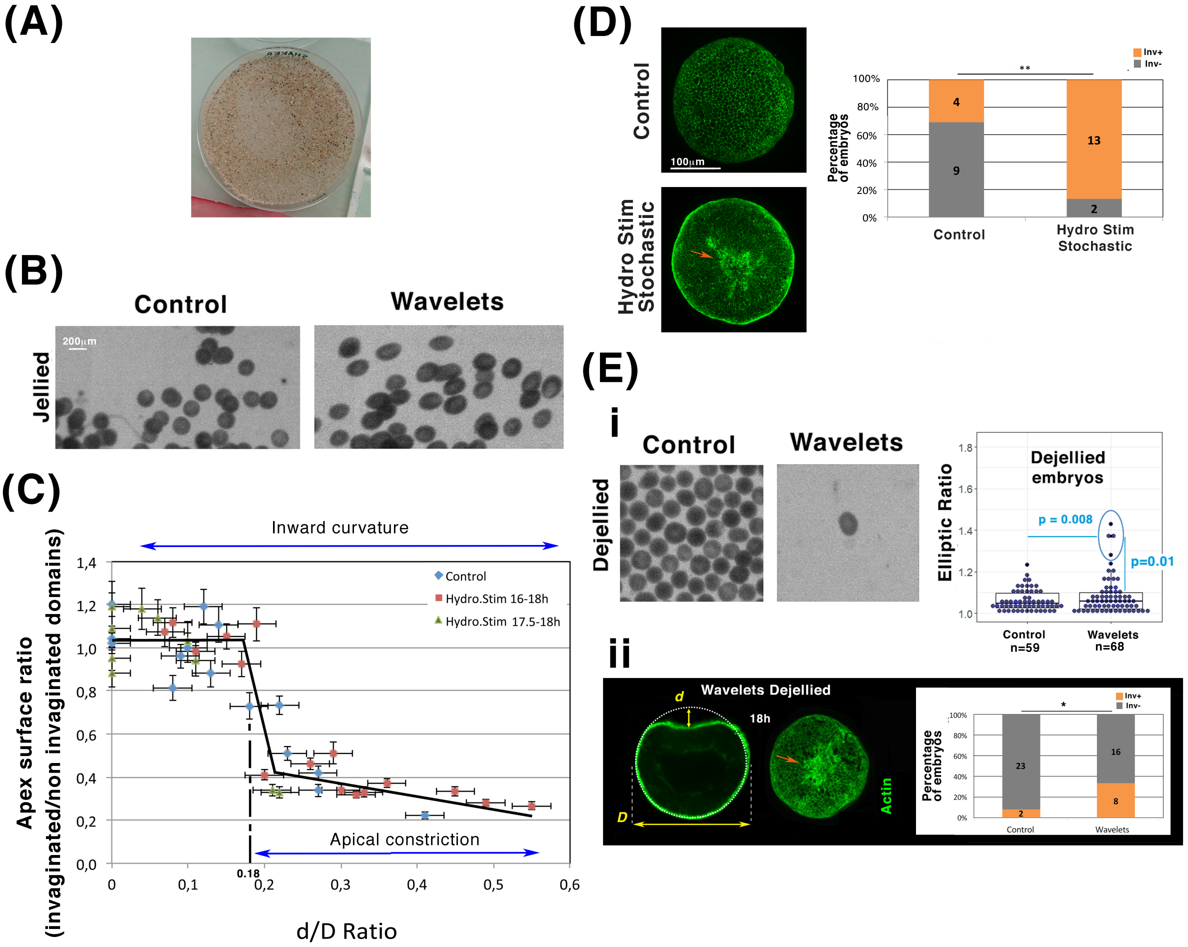

Supplementary Figure 1: *N. vectensis* hydrodynamic stimulation conditions: (A) Substrate of hydrodynamic stimulation with sand glued on pdms ground. (**B)** Embryos deformation in jelly. (**C)** Mean surface of the apex in the maximal inward invaginating domain of the embryos divided by outside of the invaginating domain, as a function of the invagination depth *d* divided by the embryo size *D* (control and stimulated embryos). Strictly up d/D=0.18 apexes are constricted. Horizontal error bars are *se*, and lateral error bars are +-2.5%. (**D)** Embryos stimulated stochastically. Orange arrows: invaginations. Scale bar is 100µm. Quantitative characterization: $n_{Control}$=13 and $n_{Wavelet}$=15 p=6.10^{-3}, (N=2 biological replicates, Fisher statistical test). (**E) i** Deformation of un-jellied embryos, and quantification (Mann-Whitney statistical test). **ii** Gastrulation of embryos submitted to hydrodynamic stimulation (N=2 biological replicates), and quantification based on significant normalized invagination depth *d/D* measurement higher or equal to 20% (see text), $n_{Control}$=25 and $n_{Wavelet}$=24 p=0.037. Fisher statistical test).

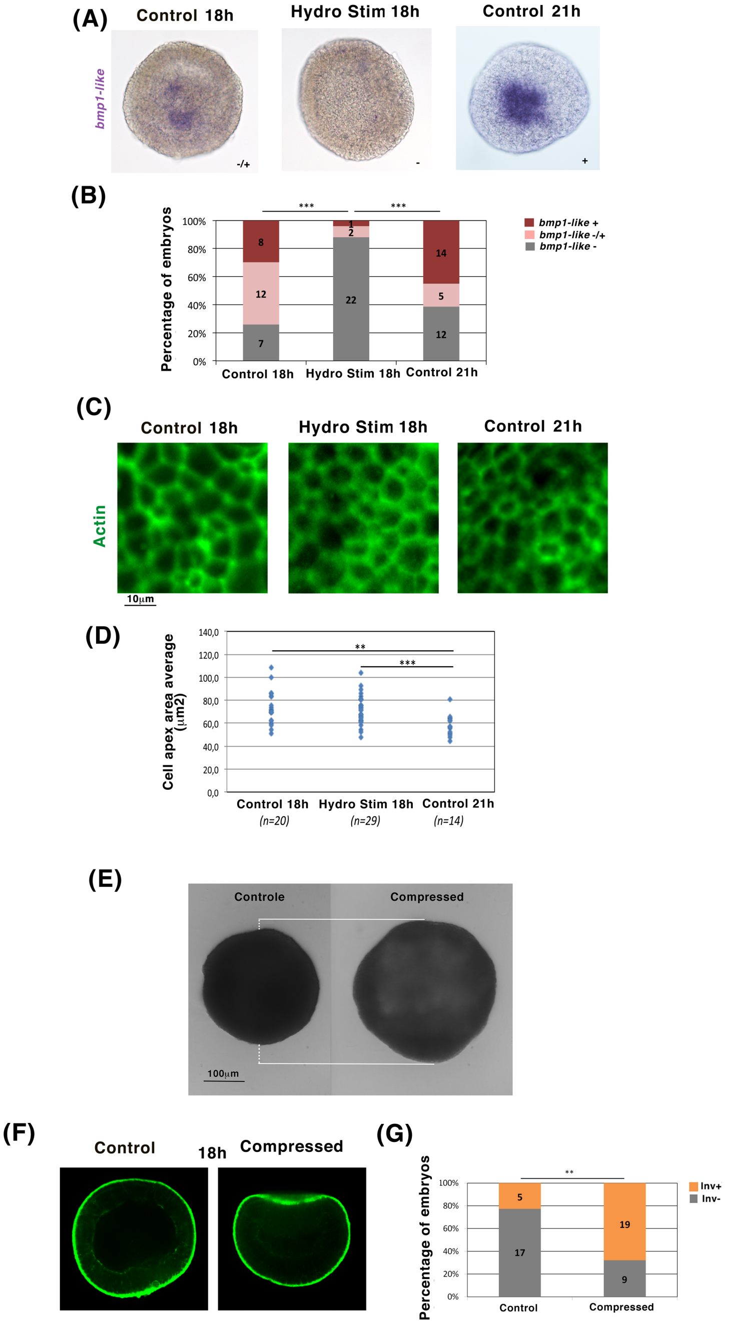

Supplementary Figure 2: (A,B) ***bmp1-like*** **expression is not stimulated by hydrodynamic stimulation. (A)** Expression of *bmp1-like* in 18h and 21h control embryos, and of 18h hydrodynamically stimulated embryos. **(B)** Quantitative analysis with no increase, rather a decrease, of *bmp-1like* expression under hydrodynamic stimulation. $n_{Control18h}$=27 and $n_{HydroStim18h}$=25 p=6.5.10^{-6}, $n_{HydroStim18h}$=25 and $n_{Control21h}$ =31 2.6.10^{-4}. Statistical test: Fisher. N=2 biological replicates. **(C,D) Mean apex cell size out of the invaginating domain does not change after hydrodynamic stimulation. (C)** Mean apex cell size in 18h controls, 18h hydrodynamically stimulated and 21h controls embryos. **(D)** Quantitative analysis. $n_{Control18h}$=20 and $n_{Control21h}$=14 p=0.0016, $n_{Control18h}$ =20 and $n_{HydroStim18h}$=29, p=0.79. Statistical test: Man-Whitney. **(E-G) Direct global uniaxial deformation stimulates gastrulation. (E)** Representative deformation of embryos by uni-axial soft compression **(F)** 18h control and 18h submitted to uniaxial compression. **(G)** Quantitative analysis. $n_{Control}$=22 and $n_{Compressed}$=28 p=2.10^{-3}. Statistical test: Fisher. N=2 biological replicates.

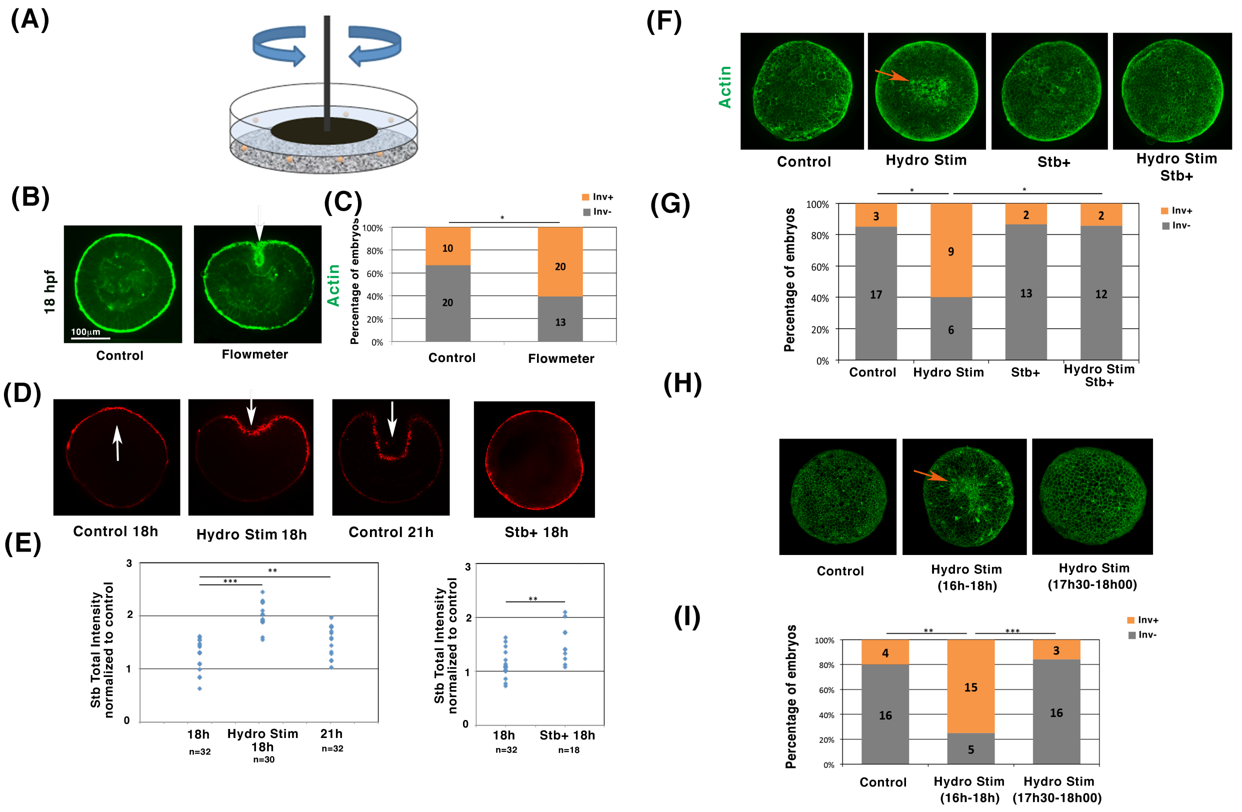

Supplementary Figure 3: (A-C) Hydrodynamic stimulation of *Nematostella* embryos gastrulation by a flow-meter. (A) Scheme of the flow-meter set-up. **(B)** Flow-meter induced gastrulation of 18h *N. vectensis* embryos. **(C)** Quantification, $n_{Control}$=30 and $n_{Flowmeter}$=33 p=0.044. Fisher statistical test. N=2 biological replicates. **(D-G) Role of Strabismus. (D)** Expression of Strabismus in 18h and 21h WT embryos, in 18h hydrodynamically stimulated embryos, and in 18h embryos injected with *strabismus* RNA (Stb+). **(E)** Quantification, Man-Whitney statistical test. N=2 biological replicate. **(F)** 18h hydrodynamically stimulated *N.vectensis* embryos injected with *strabismus* RNA (Stb+). Control 18h: injected with water. **(G)** Quantification, Fisher statistical test. N=2 biological replicates. **(H)** *N.vectensis* embryos hydrodynamically stimulated from 17h30 to 18h00. **(I)** Quantification, Fisher statistical test. N=2 biological replicates.

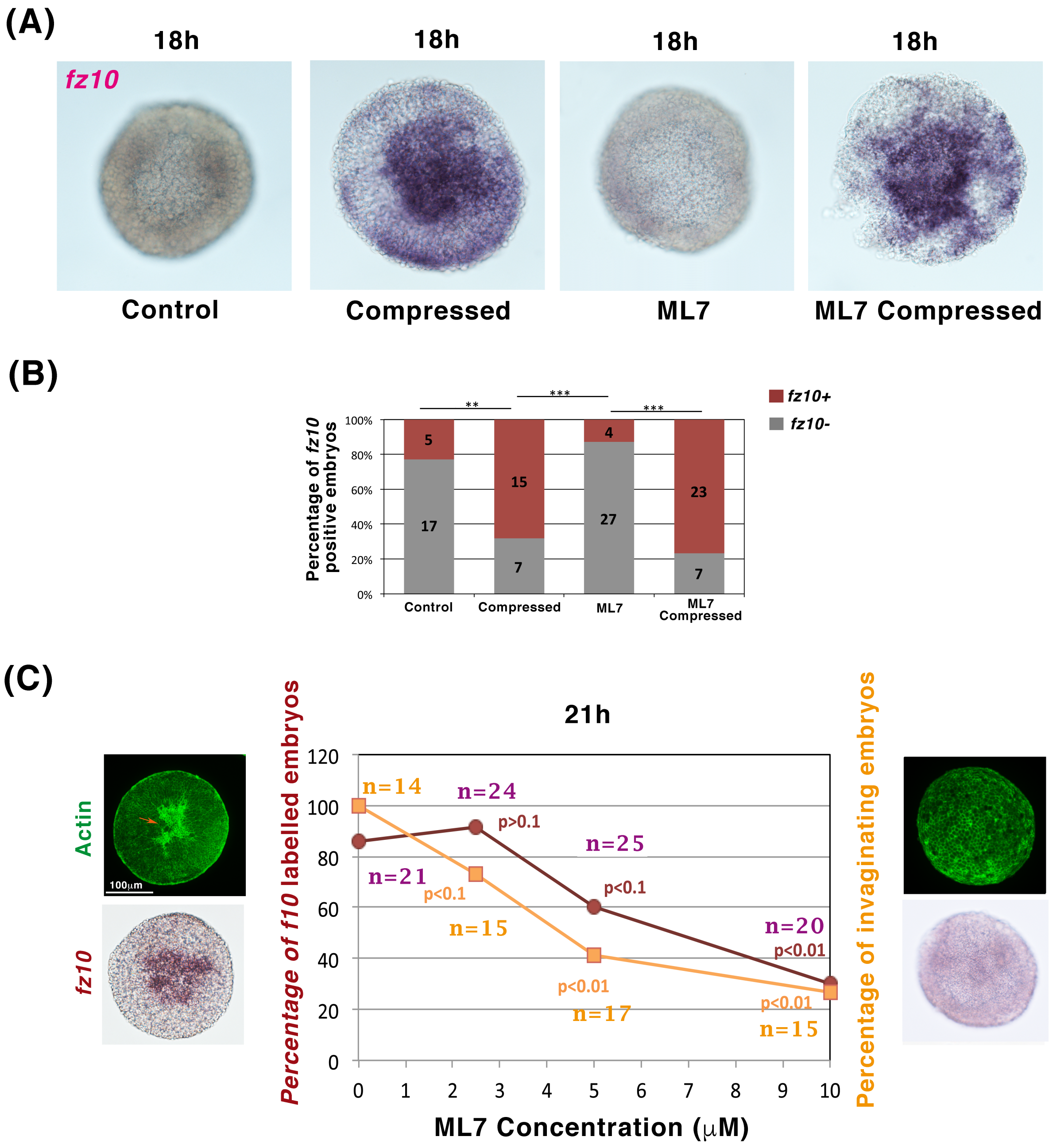

Supplementary Figure 4: Mechanical induction of *fz10* is not prevented by ML7 pharmacological treatment *per se*. (**A**) *fz10* expression in control (ethanol vehicle of ML7), ethanol vehicle compressed, ML7 and ML7 compressed embryos. (**B**) Quantitative analysis. $n_{Control}=22$ and $n_{Compressed}=22$ $p=5.8.10^{-3}$, $n_{Compressed}=22$ and $n_{ML7}=31$ $p=8.7.10^{-5}$, $n_{ML7}=31$ and $n_{ML7Compressed}=30$ $p=4.9.10^{-7}$. Statistical test: Fisher. N=2 biological replicates. (**C**) Percentage of embryos showing invagination (orange arrow) and *fz10* expression as a function of ML7 concentration**.** Fisher statistical test (comparison with control without ML7).

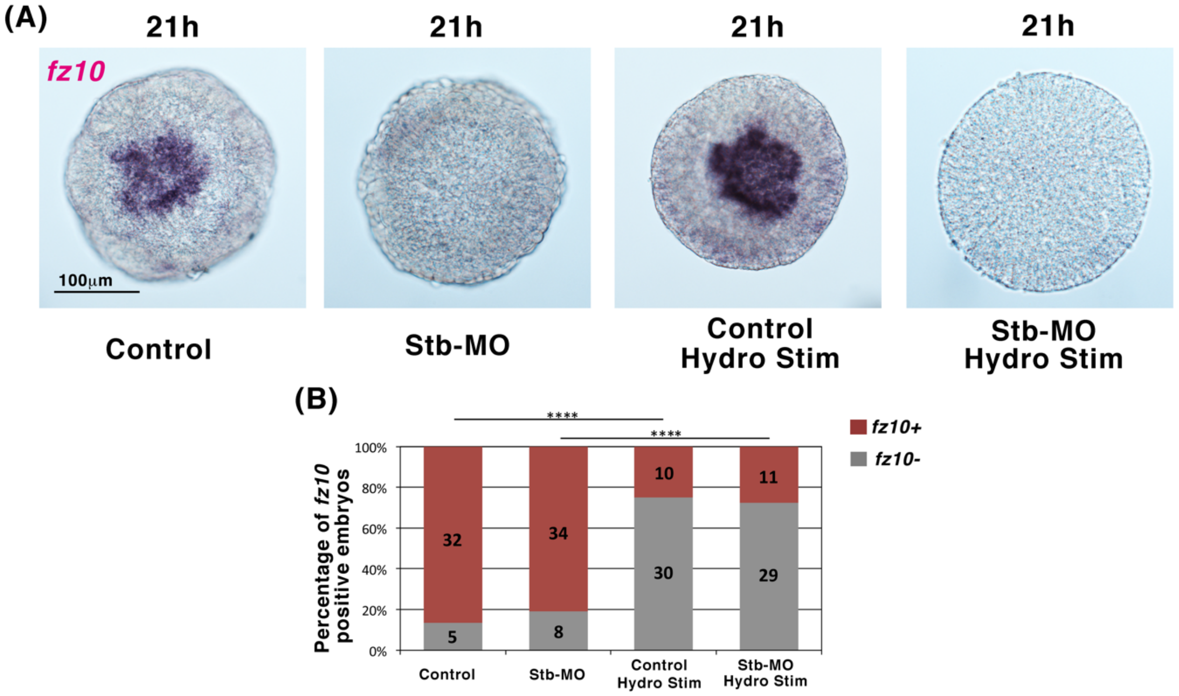

Supplementary Figure 5: Mechanical induction of *fz10* requires *stb*-dependent hydrodynamic induction of gastrulation, and is not prevented by ML7 per se. (A) *fz10* expression in water injected control, *stb-MO* injected, water injected hydrodynamically stimulated and *stb-MO* hydrodynamically stimulated embryos. (**B)** Quantitative analysis. $n_{Control}$=37 and $n_{ControlHydroStim}$=40 $p=5.4.10^{-8}$, $n_{Stb\text{-}MO}$=42 and $n_{Stb\text{-}MOHydroStim}$=40 $p=1.8.10^{-6}$. Statistical test: Fisher. N=2 biological replicates.

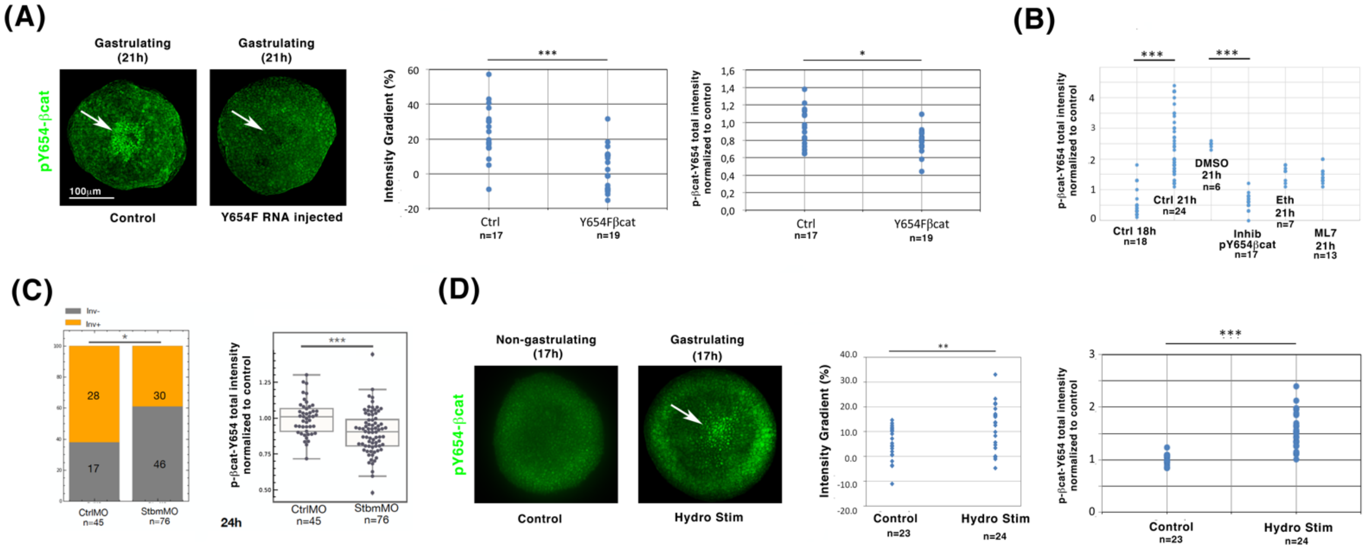

Supplementary Figure 6: Total pY654βcat levels quantification. (A) pY654-βcat labelling in 21h WT embryos, and in 21h embryos injected with the un-phosphorylable Y654F-βcat dominant negatives RNA of *N. vectensis* βcat, with quantification of the maximal gradient ratio in percentage in invaginating domains compared to non-invaginating domains and of the total intensity. Statistical test: Mann-Whitney. N=2 biological replicates. $n_{Control}$=17 and $n_{Y654F\beta cat}$=19 p=3.10^{-4}**. (B)** Quantification of total intensity of 18h non-gastrulating and 21h gastrulating *N. vectensis* embryos pharmacologically treated with p-Y654βcat inhibitor and ML7 Myo-II inhibitor**.** Values are normalized to the 18h control for total intensity. Statistical test: Mann-Whitney. N=2 biological replicates. **(C)** 24h stb-MO injected embryos defective in gastrulation ($p<0.05$, exact Fisher test). Normalised pY654-βcat cytosolic intensity analysed with computational programming (see Material & Methods). Statistical test for pY654-βcat intensity: Mann-Whitney $p<10^{-3,}$ N=3 biological replicates. **(D)** pY654-βcat labelling in *N. vectensis* embryos gastrulating in response to hydrodynamic stimulation from 16h to 17h, with quantification of the maximal gradient ratio in percentage in invaginating domains compared to non-invaginating domains and of the total intensity. $n_{Control}$=23 and $n_{HydroStim}$=24 p= 2.4.10^{-2}. Statistical test: Mann-Whitney. N=2 biological replicates.

Supplementary information

Sup Info 1:

Marine streams can be generated both by the tide or by temperature gradients (Prants et al., 2018). Tide induced streams can reach $m.s^{-1}$ velocities at surface, a velocity progressively modulated down to 0 $m.s^{-1}$ every 6 h as a function of time, as well as progressively down modulated by depth and by local protection by rocks inhomogeneities undersea or on the shore. Regimes on the order of magnitude of $10cm.s^{-1}$ are thus included in the large panel of velocities regime ensured by both tide periodicity, marine depth variability and rock inhomogeneities, with a stability of a couple of hours ensured by the 6 h tides periodicity. Adding to tide streams, temperature gradient induced permanent streams ensure a stable stream on the order of $10cm.s^{-1}$ on the shore (Prants et al., 2018). In addition to these streams, waves on the shore lead to velocities stable for a couple of hours, that can also range from order of magnitudes of $1m.s^{-1}$ (for 10s long period waves) to 10 $cm.s^{-1}$ (for 2s low period waves) (associated to the two frequencies found Fig.4 of ref (Chang et al., 2017)). Here we thus concentrated on the 10 $cm.s^{-1}$ velocities, shared by both temperature gradient induced streams and soft waves on the shore regimes. We furthermore focused on 2s periodic regimes characteristic of environmental soft 10 $cm.s^{-1}$ waves on the shore to test their mechanotransductive ability to trigger Myosin-dependent induction of gastrulation in *Nematostella*.

As the flow induced by waves on the shore can change as a function depth, wind-meteorologic and geographic conditions, this thus tests the wave induced inductive power to simulate *Nematostella* embryos gastrulation in a given but frequently encountered soft waves regime.

The temperature of oceans has necessarily changed between 2 billion years ago and today. However, cells necessarily adapted to temperature changes. Thus, applying the 2 billion to 700 million years ago temperature of oceans to the species here tested that are living in around 20°C temperature today, would necessarily strongly perturb their physiology. Temperature was thus kept at the current temperature at which these are leaving species today.

Sup Info 2:

A- The d/D>18% or d/D>=20% threshold is the minimum value from which is observed apical constriction in the invagination compared to non-invaginating domains, characterizing when curvature deformation is associated to apex constrictions and can be considered as a gastrulation initiation (Supplementary Fig.1C).

B- Dejellied embryos were hydrodynamically stimulated following the same procedure that jellied embryos, except that sand was removed as individual dejellied embryos could stay immobilized between sand grains during stimulation. The deformation of the blastulae was monitored by fast imaging, and also found to be elliptic (Supplementary Fig.1E-i). The elliptic deformation of the embryos (long axis over short axis L/W) was found to be of 25-40% for 7% of the embryos at the given time of their photograph (Supplementary Fig.1E-i-right-blue ring - $n_{Control}$=59 and $n_{Wavelet}$=68, p=0.008).

As seen in Supplementary Fig.1E-ii-hydrodynamically stimulated ($n_{Control}$=25 and $n_{Wavelet}$=22, p=0.037), we observed that gastrulation was initiated at 18h in nearly 30% of the stimulated embryos with a normalized *d/D* invagination depth larger than 18% with observation of apex constriction in the invaginating tissue (Supplementary Fig.1E-ii, orange array), in contrast to static control in which only 8% of the embryos have initiated gastrulation.

The gel radius (1.5 to 4 mm) is 10 times greater than the *N. vectensis* embryo radius (~ 0.1mm radius). As Stokes forces are proportional to the radius, we expect the forces to be 10 times greater on the gel than on the dejellied individual embryos. Assuming an elastic modulus for the gel of the same order of magnitude than for embryos, one expects a deformation of embryos significantly more important in jellied embryos, than in un-jellied embryos, in addition to potential sand friction effects on the ground. This is observed in Fig.1C and explains the higher rate of gastrulation response of jellied embryos experiments compared to de-jellied embryos experiments. This indicates, in addition, that deformation forces and not chock forces (that are absent in jellied embryos) triggers gastrulation.

Note that, unless todays *N. vectensis* embryo's natural environment may differ from ancient embryos environment, here we are testing on dejellied *N. vectensis* embryos, in addition to jellied embryos, the plausibility of an evolutionary conservation of a mechanical stimulation of its gastrulation by hydrodynamic stimulations that can be found on the sea shoreline.

C- Interestingly, if *bmp-1* expression was not increased by hydrodynamic stimulation, it was found to be decreased. The fact that a β-cat independent gene expression, like *bmp-1*, could be downregulated mechanically interestingly suggests that other embryonic patterns might be mechanosensitive via other mechanosensitive pathways, an object of future investigations.

D- Tension can be applied by different means, therefore gastrulation can be induced by different ways that can generate tension. The important point here is to test if the most frequent natural mechanical environment of marine embryos (hydrodynamic flow) is efficient enough in triggering gastrulation within natural marine environmental conditions, independently of the fact that other less frequent type of mechanical stimulation (like a direct mechanical compression) can trigger it.

Sup Info 3:
A- ML7 is specific of MLCK at 10μM (https://www.abcam.com/ml7-myosin-light-chain-kinase-inhibitor-ab120848.html) and Blebbistatin is specific of Myo-II at 50 μM (Kovacs et al., 2004). Latrunculin B targeting Actin was toxic from the beginning of development and could thus not be tested. Myo-II expression by morpholino injection would prevent embryonic development, which is the reason why pharmacological inhibition was chosen. The use of two pharmacological inhibitors targeting two distinct proteins required for Myo-II activity, ensures the specificity of the Myo-II activity tested here in hydrodynamically induced invagination.
B- To our knowledge, no Myosin-II antibody was found to efficiently label *N. vectensis* Myo-II, preventing us to also follow the Myo-II behavior in *N. vectensis*.

Sup Info 4:
A- Because injections can only be done in dejellied embryos, the role of *stb* in hydrodynamic stimulation of gastrulation initiation was tested on dejellied embryos.
B- In *stb-MO* injected embryos, hydrodynamic stimulation was performed at developmental stage when *stb* was characterized as required for gastrulation initiation (Kumburegama et al., 2011), namely between 19h and 21h here at 18°C.
C- Note that at later stages of development, 28h, an additional and Myo-II-independent process appears to participate in *N. vectensis* invagination forces (Pukhlyakova et al., 2018).

Sup Info 5:
To test whether ML7, or Myo-II inhibition in themselves, could block *fz10* expression independently of mechanical stress, we submitted the ML7 treated embryos to direct uni-axial mechanical deformation from 16h to 18h. In contrast to the inhibition of *fz10* expression induction in hydrodynamically stimulated embryos in which mechanical morphogenetic movements of gastrulation was impaired by ML7 treatment (Fig.3D,F), *fz10* expression was stimulated in the presence of ML7 when embryos were mechanically deformed directly by nearly 20% (Supplementary Fig.4A,B with an enhancement of a factor of nearly 7 of this percentage in response to strain). ML7 and associated Myo-II inhibition can thus not *per se* inhibit *fz10* expression in the presence of a mechanical strain.
Note that uni-axial deformation that necessary leads to an internal pressure increase should lead to higher mechanical epithelial tissue stress than hydrodynamically induced shear stress inducing by definition of shear stress no internal pressure, on non-invaginating embryos. This explains the *fz10* response of uni-axially compressed ML7 treated embryos compared to the non-response of hydrodynamically shear stressed ML7 treated embryos.
We thus propose that the Myo-II-dependent tension in the constricting and invaginating domain is enough to trigger the mechanical induction of the β-cat target gene *fz10* in the gastrulating domain, but that the hydrodynamically induced shear stress deformation of the overall embryo is not sufficient to trigger *fz10* ectopically into the ectoderm out of the gastrulating domain in hydrodynamically strained embryos (Fig.3C,E). We propose that in contrast, *fz10* is also found to be expressed ectopically in the ectoderm out of the gastrulating domain in embryos compressed uni-axially with a coverslip (Supplementary Fig. 4A,B), due to the higher tension in the epithelium of uni-axially compressed embryos compared to embryos shear stressed deformed by hydrodynamic stimulation, similarly to the β-cat target gene *brachyury* also observed as ectopically expressed in response to uniaxial deformation in addition to its natural pattern of expression in *N. vectensis* (Pukhlyakova et al., 2018).

Sup Info 6:
In contrast to reference (Kumburegama et al., 2011), in which *fz10* shows partial defects in *stb-MO* injected embryos, here we find that *fz10* expression is more strongly defective in *stb-MO* gastrulating defective embryos. This difference could be associated to differences in the concentration of the *stb-MO* injected potentially leading to differences of *stb*-dependent internal stresses.

Sup Info 7:
Based on the strong conservation of the Y654 site of βcat in Metazoa including *N. vectensis* (Roper et al., 2018), anti-bodies against the human phosphorylated form of βcat (pY654βcat) were here used in *N. vectensis* (see Methods), showing labelling in the gastrulating domain of *N. vectensis* embryos in gastrulating embryos at 21h, in contrast to 18h non-gastrulating embryos (Fig.4A,B). Western blot confirmed the specific labelling of the pY654βcat in *N. vectensis* observed at 21h and not at 18h with the anti-body (Fig.4A,B). Further confirming specificity *N. vectensis* eggs injected with the RNA encoding the dominant negative form of βcat Y654F (Somogyi and Rorth, 2004) showed no pY654-βcat labelling observed in 21h gastrulating *Nv* embryos (see Materials and methods, Supplementary Fig.6A).

Sup Info 8:

Because the overall levels of pY654-βcat increases in the embryo, the animal-vegetal signal ratio of pY654-βcat increase cannot be interpreted by a pY654-βcat decrease in the vegetal domain of the embryo and is due to an increase in pY654-βcat in the oral EM (Supplementary Fig.6B).

Sup Info 9:

Injecting *stb-MO*, that has previously been described to prevent *N. vectensis* gastrulation (Kumburegama et al., 2011), decreased Y654-βcat phosphorylation total levels (as detected with cell scale imaging analysis) correlatively to the partial decrease in the number of invaginating and constricting embryos at 24h (the time at which *stb-MO* phenotypes most clearly distinguishes from WT phenotypes) (Merle, 2018), consistent with Y654-βcat phosphorylation sensitivity to gastrulation found in gastrulation defective ML7 treated embryos (Supplementary Fig.6C).

Sup Info 10:

Mechanical induction of Y654-βcat phosphorylation can be transient (Brunet et al., 2013), and could here be transiently detected after 1 hour of hydrodynamically stimulated gastrulation (*i.e* at 17h) only, in *N. vectensis* (Supplementary Fig.6D).

Sup Info 11:

A- Note that DMSO perturbed the expression of *fz10* in 18h embryos, such that S381-0393 was used directly in sea water.

B- Note that unless injection *per se* reduced the proportion of *fz10* positive embryos in both 18h hydrodynamically stimulated and 21h embryos, Y654Fβcat RNA injection almost fully inhibited the proportion *fz10* positive embryos in the invagination in both cases, in a statistical relevant way.

C- At gastrulation stage, β-cat nuclear staining was localized in single nuclear and peri-nuclear dots, co-localized with Dapi, as well as sometimes found as condensed out of the nucleus, as observed in other systems (Fig.4 E-H) (Zhang et al., 2014).

Sup Info 12:

Removing light sensitive induction of inversion might potentially have a role in the mechanical induction of inversion. However, light sensitive reversion *of C .flexa* is a Myo-II dependent process of inversion which is induced by a transition from light to darkness (Brunet et al., 2019). Under light, *C .flexa* is thus inactive. All of our experiments are performed under light, thus removing light sensitivity does not inactivate *C. flexa* that are already inactive in their initial state (before mechanical stimulation), which should not introduce any perturbation before and during the mechanical stimulation of inversion under light.

Sup Info 13:

A- Fixation and labelling procedures on hydrodynamically stimulated colonies led to small fragments of the inverted *C. flexa* partially or fully closed on themselves in a rod-like flagella-out shape by hydrodynamic stimulation.

B- *C. flexa* colonies were submitted to hydrodynamic flow for 2 minutes in the presence of 200nm fluorescence particles, then left static for one hour. Fluorescent particles internalisation, that reflect *C. flexa* cells feeding (Brunet et al., 2019), was found to be nearly 2 times more efficient in

the inverted closed *C. flexa* than in the identically agitated non-inverted ones. This suggests a feeding process enhanced by particles trapping by phagocyting cells, due to *C. flexa* hydrodynamically induced inversion enclosing particles found in solution by hydrodynamic flow.
C- Note that Myosin activation is known to be involved in single cell phagocytosis in specialised cells such as macrophages (Tsai and Discher, 2008). In *C. flexa*, light induced activation of Myo-II leading to partial (unclosed) inversion does not lead to feeding efficiency increase (Brunet et al., 2019) (see below). This indicates that the increase of *C. flexa* feeding efficiency after mechanical stimulation of Myo-II dependent full inversion cannot be attributed to a Myo-II dependent increase of phagocytosis rate in individual cells.
D- Note that light induced partial inversion leads to a decrease of the inverted and opened *C. flexa* feeding efficiency, suggested as due to cell packing constraints by inversion that reduces phagocytosis efficiency (Brunet et al., 2019). Here we find an increase of feeding efficiency in fully closed inverted *C. flexa,* overcoming the latter packing effect, which we suggest being due to the trapping of nutriments put in suspension by the flow, by the full closing of *C. flexa* stimulated by the same flow.

Sup Info 14:
The *Ephydatia muelleri* β-cat and *N.vectensis fz10* sequence were compared to predictive coding sequences of *C.flexa* (Brunet et al., 2019) with BLAST. A maximum of 22.6% identity and 51.7% similarity only was found for β-cat, without the Y654-β-cat motif conservation characteristic of all Metazoa (Roper et al., 2018), and of 30% of similarity only was found for *fz10* (not shown).

Sup Info 15:
Hydrodynamic mechanical strains are not present in the environment of insects bilaterian gastrulating embryos like Drosophila embryos. They are neither required for endogenous gastrulation of a Cnidaria embryo like *Nematostella* embryos (that initiate gastrulation at 21h of development independently of any flow). Therefore, it does not appear probable that a common hydrodynamic environment was at the origin of mechanical induction of gastrulation emergence several times in a convergent-evolution process, in the Drosophila and *N. vectensis* that are representatives of the evolutionary distant Bilateria and Cnidaria superphyla. In contrast, these distinct evolutionary distant species show a common response of Myosin-dependent tissue curvature inversion process to environment mechanical cues with multi-cellular *C. flexa* choanoflagellate representatives that are relevant to the marine environment of their last common Metazoa ancestors. Thus, it is more probable that strains reminiscent of the mechanical environment of their common ancestor here reactivate a fossil behavior of gastrulating in response to hydrodynamic marine strains, than to have remerged several times during evolution though a convergent-evolution process.

An alternative approach to this reasoning would be that, independently of hydrodynamic or other environmental mechanical cues, embryos have developed abilities to develop their own internal mechanical strains. For instance, these could have been developed through cell division in a closed embryo, or through active cell shape changes. However, even though this is the case for Drosophila embryos, in which *snail*-dependent internal mechanical strains of pulsations mechanotransductively activate Myo-II-dependent gastrulation (Mitrossilis et al., 2017), this is not the case for *C.flexa*.

Sup Info 16:
The induction of EM cell differentiation in hydrodynamically-induced gastrulating tissues of the Cnidarian *N.vectensis* 18h embryos is due to the mechanical stimulation of the phosphorylation of Y654-βcat, leading to its translocation to the nucleus and to the transcription of *fz10* (Fig.4). This feature is shared with early Drosophila and zebrafish embryos, and human embryonic stem cells in which mechanical strains (in these cases not hydrodynamically stimulated) lead to endo-mesodermal gene expression (*twist* (Farge, 2003, Shorr et al., 2019), *brackury* (Brunet et al., 2013), and *BMP4* (Muncie et al., 2020) respectively) downstream of mechanically induced Y654-βcat phosphorylation as well (Brunet et al., 2013, Muncie et al., 2020).

In Drosophila embryos, the underlying mechanistic process of translation of a mechanical strain into a biochemical reaction leading to Y654-βcat phosphorylation consists in the mechanically induced increase of probability of the opening of the Y654-βcat/D665-Ecadh major site of interaction of the βcat/Ecadh complex at junctions under stress, that increases the accessibility of Y654-βcat to Src family kinases phosphorylation (Roper et al., 2018). Once phosphorylated, Y654-βcat can no more interact with D665-Ecadh: the βcat is thus released from junctions and enriches the cytosol, stimulating Twist expression once in the nucleus (Desprat et al., 2008).

In *Nematostella* embryos, the Y654-βcat phosphorylation by mechanical strains shared with Drosophila and zebrafish embryos might be explained by a similar underlying mechanistic process, or by alternative mechanisms, involving for instance a change of biochemical environment (for instance morphogen gradients internal or external to the embryo, like in intestinal villi (Shyer et al., 2015)) of the tissue due to invagination shape change at the tissue level, and leading to the Y654-βcat phosphorylation upstream of *fz10* expression, independently of a shape change occurring at the molecular level through the βcat/Ecadh complex change of conformation.

Interestingly, such alternative mechanism would still remain a mechanically induced EM differentiation process, because a mechanical tissue shape change (curvature inversion) would induce EM differentiation, in a given biochemical context allowing it (a morphogen gradient). Indeed, in the case of this alternative hypothesis, the biochemical context would be a morphogen gradient able to activate a pathway leading to Y654-βcat phosphorylation at certain concentration of signaling proteins perceived by epithelial cells due to the inward curvature deformation of the epithelium at gastrulation, namely due a mechanical shape change at the tissue level. In Drosophila embryos, the biochemical context is the presence of already activated Src-familly kinases able to phosphorylate Y654-βcat (Src42A) once the site made accessible by a conformation change (Desprat et al., 2008, Roper et al., 2018), namely due to a mechanical shape change at the molecular level.
Therefore, unless the underlying mechanistic process of translation of a mechanical deformation into the biochemical reaction leading to Y654-βcat phosphorylation in distinct species might possibly be different with a mechanical deformation input acting at distinct scales (the tissue scale v/s the molecular scale), mechanical induction of Y654-βcat phosphorylation leading to endo-mesoderm gene expression would remain a feature shared by bilaterian and cnidarian representatives.

Would such diversified underlying mechanistic processes be at work in the mechanical induction of Y654-βcat phosphorylation by tissue curvature inversion in distinct species, this could interestingly suggest a convergent-evolution process having selected mechanically inducted Y654-

βcat phosphorylation. The latter process would have led to EM genes expression in tissues undergoing curvature inversion as a favorable feature (like division of labor introduced by the existence of at least two cell types into the same multicellular primitive organism (Ruiz-Trillo and de Mendoza, 2020, Cavalier-Smith, 2017)) independently in different Metazoa phyla during the course of evolution, thought distinct underlying mechanistic processes.
On the other hand, as far as βcat including an Y654 site able to interact with E-cadherin has emerged concomitantly with multicellular metazoan emergence in the course of evolution (Fernandez-Sanchez et al., 2015), and given the presence of phosphorylating kinases in all metazoan so probably from first metazoan (Miller, 2012), multi-cellular tissues cannot escape a mechanically induced opening of Y654-βcat under strain leading to its phosphorylation. And to the expression of any target genes (as the TCF co-transcription factor of βcat is present in all metazoan and thus probably from first metazoan as well (Cadigan and Waterman, 2012)). Which should thus be acting in response to gastrulation of first metazoan, and be conserved from first metazoan *per se*.

This does not prevent the existence of *additional* mechanical induction and biochemical induction processes having possibly diversified and re-enforced it in distinct phyla *a posteriori* during the course of evolution. Deciphering all underlying mechanistic processes involved in the translation of a mechanical deformation into the biochemical reaction leading to Y654-βcat phosphorylation in a given species, here *N.vectensis*, is a full project in itself (see reference (Roper et al., 2018)) for future investigations.

Sup Info 17:

The conditions that led to the origin of gut development in Metazoa, that is intimately associated to animal development origins, are a persisting opened question of evolution (Cavalier-Smith, 2017, Ruiz-Trillo and de Mendoza, 2020). The presence of bacteria *per se* was proposed to have favored first transient forms of small multicellular rosette closed aggregates that trap bacteria in Choanoflagellates, the closest living single celled sister group of Metazoa (Dayel et al., 2011). These structures, inherently coupled to feeding functions, are considered as possible fossils of conditions that led to the emergence of multi-cellularity in pre-Metazoa (Dayel et al., 2011). However, once having evolved in stabilized epithelial-like multicellular sheets, a pre-Metazoa tissue must maintain the property to trap bacteria and nutriments for feeding. Flagellate movements *per se* were suggested to induce a flow that favors attraction of bacteria at the phagocyting heads of multi-cellular choanoflagellates (Brunet et al., 2019). Here we find that hydrodynamically induced inversion rescues trapping conditions, in a Myo-II dependent process possibly re-enforced by Myo-II expression mechanical induction. Thus, it enhances feeding efficiency of nutriments found in suspension due to the flow. This suggests that hydrodynamically induced Myo-II dependent tissue curvature inversion ability of initially un-closed stabilized multicellular pre-Metazoa could have been favorably selected though evolution, and conserved in first Metazoa through hydrodynamic stimulation of gastrulation.

Note that Drosophila and *N.vectensis* embryos are not known to gastrulate or invert in a light sensitive way. This suggest that the light sensitivity of *C.flexa* inversion, which we here inhibited, was indeed not present in the common ancestor of Drosophila, *N.vectensis*, *C.flexa* and was probably acquired by *C.flexa* during the course of evolution.

Spontaneous amplification of cell differentiation and partitioning functions allowed by multi-cellularity were suggested to be at the origin of first Metazoa tissue differentiation (Newman, 2020). Moreover, it was speculated that attachment of closed rosette-like colonies of pre-

Metazoa multi-cellular tissues with physical substrates may be due to the first cell differentiations between substrate-adherent and non-adherent cells to form pre-sponge tissues (Cavalier-Smith, 2017). The results presented in this study suggest that the mechanical strains of inversion *per se* might have initiated the process of β-cat dependent EM differentiation in Metazoa tissues characterized by the presence β-cat/E-cadherin adherens junctions, and of β-cat target genes.

Supplementary Information References

BRUNET, T., BOUCLET, A., AHMADI, P., MITROSSILIS, D., DRIQUEZ, B., BRUNET, A.-C., HENRY, L., SERMAN, F., BÉALLE, G., MÉNAGER, C., DUMAS-BOUCHIAT, F., GIVORD, D., YANICOSTAS, C., LE-ROY, D., DEMPSEY, N. M., PLESSIS, A. & FARGE, E. 2013. Evolutionary conservation of early mesoderm specification by mechanotransduction in Bilateria. *Nat Commun,* 4.

BRUNET, T., LARSON, B. T., LINDEN, T. A., VERMEIJ, M. J. A., MCDONALD, K. & KING, N. 2019. Light-regulated collective contractility in a multicellular choanoflagellate. *Science,* 366**,** 326-334.

CADIGAN, K. M. & WATERMAN, M. L. 2012. TCF/LEFs and Wnt signaling in the nucleus. *Cold Spring Harb Perspect Biol,* 4.

CAVALIER-SMITH, T. 2017. Origin of animal multicellularity: precursors, causes, consequences-the choanoflagellate/sponge transition, neurogenesis and the Cambrian explosion. *Philos Trans R Soc Lond B Biol Sci,* 372.

CHANG, Y. S., DO, J. D., KIM, S. S., AHN, K. & JIN, J. Y. 2017. Measurement of Turbulence Properties at the Time of Flow Reversal Under High Wave Conditions in Hujeong Beach. *Journal of Korean Society of Coastal and Ocean Engineers,* 29(4)**,** 206~216.

DAYEL, M. J., ALEGADO, R. A., FAIRCLOUGH, S. R., LEVIN, T. C., NICHOLS, S. A., MCDONALD, K. & KING, N. 2011. Cell differentiation and morphogenesis in the colony-forming choanoflagellate Salpingoeca rosetta. *Dev Biol,* 357**,** 73-82.

DESPRAT, N., SUPATTO, W., POUILLE, P.-A., BEAUREPAIRE, E. & FARGE, E. 2008. Tissue deformation modulates twist expression to determine anterior midgut differentiation in Drosophila embryos. *Developmental Cell,* 15**,** 470-477.

FARGE, E. 2003. Mechanical induction of twist in the Drosophila foregut/stomodeal primordium. *Curr Biol,* 13**,** 1365-77.

FERNANDEZ-SANCHEZ, M. E., BRUNET, T., ROPER, J. C. & FARGE, E. 2015. Mechanotransduction's Impact in Animal Development, Evolution, and Tumorigenesis. *Annu Rev Cell Dev Biol.*

KOVACS, M., TOTH, J., HETENYI, C., MALNASI-CSIZMADIA, A. & SELLERS, J. R. 2004. Mechanism of blebbistatin inhibition of myosin II. *J Biol Chem,* 279**,** 35557-63.

KUMBUREGAMA, S., WIJESENA, N., XU, R. & WIKRAMANAYAKE, A. H. 2011. Strabismus-mediated primary archenteron invagination is uncoupled from Wnt/beta-catenin-dependent endoderm cell fate specification in Nematostella vectensis (Anthozoa, Cnidaria): Implications for the evolution of gastrulation. *Evodevo,* 2**,** 2.

MERLE, T. 2018. *Conservation entre Cnidaires et Bilatériens d'une voie mécanosensible induisant la formation de l'endomésoderme.* PhD Thèse de doctorat, Sorbonne Université.

MILLER, W. T. 2012. Tyrosine kinase signaling and the emergence of multicellularity. *Biochim Biophys Acta,* 1823**,** 1053-7.
MITROSSILIS, D., ROPER, J. C., LE ROY, D., DRIQUEZ, B., MICHEL, A., MENAGER, C., SHAW, G., LE DENMAT, S., RANNO, L., DUMAS-BOUCHIAT, F., DEMPSEY, N. M. & FARGE, E. 2017. Mechanotransductive cascade of Myo-II-dependent mesoderm and endoderm invaginations in embryo gastrulation. *Nat Commun,* 8**,** 13883.
MUNCIE, J. M., AYAD, N. M. E., LAKINS, J. N., XUE, X., FU, J. & WEAVER, V. M. 2020. Mechanical Tension Promotes Formation of Gastrulation-like Nodes and Patterns Mesoderm Specification in Human Embryonic Stem Cells. *Dev Cell,* 55**,** 679-694 e11.
NEWMAN, S. A. 2020. Cell differentiation: What have we learned in 50 years? *J Theor Biol,* 485**,** 110031.
PRANTS, S. V., ULEYSK, M. Y. & BUDYANSKY, M. V. 2018. Lagrangian Analysis of Transport Pathways of Subtropical Water to the Primorye Coast. *Doklady Earth Sciences,* 481**,** 1099–1103.
PUKHLYAKOVA, E., AMAN, A. J., ELSAYAD, K. & TECHNAU, U. 2018. beta-Catenin-dependent mechanotransduction dates back to the common ancestor of Cnidaria and Bilateria. *Proc Natl Acad Sci U S A*.
ROPER, J. C., MITROSSILIS, D., STIRNEMANN, G., WAHARTE, F., BRITO, I., FERNANDEZ-SANCHEZ, M. E., BAADEN, M., SALAMERO, J. & FARGE, E. 2018. The major beta-catenin/E-cadherin junctional binding site is a primary molecular mechano-transductor of differentiation in vivo. *Elife,* 7.
RUIZ-TRILLO, I. & DE MENDOZA, A. 2020. Towards understanding the origin of animal development. *Development,* 147.
SHORR, A. Z., SONMEZ, U. M., MINDEN, J. S. & LEDUC, P. R. 2019. High-throughput mechanotransduction in Drosophila embryos with mesofluidics. *Lab Chip*.
SHYER, A. E., HUYCKE, T. R., LEE, C., MAHADEVAN, L. & TABIN, C. J. 2015. Bending gradients: how the intestinal stem cell gets its home. *Cell,* 161**,** 569-580.
SOMOGYI, K. & RORTH, P. 2004. Evidence for tension-based regulation of Drosophila MAL and SRF during invasive cell migration. *Dev Cell,* 7**,** 85-93.
TSAI, R. K. & DISCHER, D. E. 2008. Inhibition of "self" engulfment through deactivation of myosin-II at the phagocytic synapse between human cells. *J Cell Biol,* 180**,** 989-1003.
ZHANG, M., MAHONEY, E., ZUO, T., MANCHANDA, P. K., DAVULURI, R. V. & KIRSCHNER, L. S. 2014. Protein kinase A activation enhances beta-catenin transcriptional activity through nuclear localization to PML bodies. *PLoS One,* 9**,** e109523.
